# Single-cell transcriptomic analysis reveals differential cell subpopulations and distinct phenotype transition in normal and dissected ascending aorta

**DOI:** 10.1186/s10020-022-00584-4

**Published:** 2022-12-19

**Authors:** Yu-bin He, Hai-zhen Jin, Jin-long Zhao, Chong Wang, Wen-rui Ma, Jie Xing, Xiao-bin Zhang, Yang-yang Zhang, Huang-dong Dai, Nai-shi Zhao, Jian-feng Zhang, Guan-xin Zhang, Jing Zhang

**Affiliations:** 1 Department of Cardiovascular Surgery, Shanghai Chest Hospital, Shanghai Jiao Tong University, No.241, West Huaihai Road, Shanghai, 200030 China; 2 Department of Central Laboratory, Shanghai Chest Hospital, Shanghai Jiao Tong University, Shanghai, China; 3 Department of Cardiovascular Surgery, Shanghai Jiao Tong University Affiliated Sixth People’s Hospital, Shanghai, China; 4 Department of Cardiac Surgery, Zhongshan Hospital, Fudan University, Shanghai, China; 5 Department of Biobank, Shanghai Chest Hospital, Shanghai Jiao Tong University, Shanghai, China; 6 Department of Cardiothoracic Surgery, Changhai Hospital, Second Military Medical University, No.168, Changhai Road, Shanghai, China

**Keywords:** Single-cell RNA sequencing, Acute thoracic aortic dissection, Cell differentiation trajectory, CXCL12, Cell–cell interaction, Phenotypic switch

## Abstract

**Background:**

Acute thoracic aortic dissection (ATAD) is a fatal condition characterized by tear of intima, formation of false lumen and rupture of aorta. However, the subpopulations of normal and dissected aorta remain less studied.

**Methods:**

Single-cell RNA sequencing was performed including 5 patients with ATAD and 4 healthy controls. Immunohistochemistry and immunofluorescence were used to verify the findings.

**Results:**

We got 8 cell types from human ascending aorta and identified 50 subpopulations including vascular smooth muscle cells (VSMCs), endothelial cells, fibroblasts, neutrophils, monocytes and macrophages. Six transmembrane epithelial antigen of prostate 4 metalloreductase (STEAP4) was identified as a new marker of synthetic VSMCs. CytoTRACE identified subpopulations with higher differentiation potential in specified cell types including synthetic VSMCs, enolase 1^+^ fibroblasts and myeloid-derived neutrophils. Synthetic VSMCs-derived C-X-C motif chemokine ligand 12 (CXCL12) might interact with neutrophils and fibroblasts via C-X-C motif chemokine receptor 4 (CXCR4) and atypical chemokine receptor 3 (ACKR3), respectively, which might recruit neutrophils and induce transdifferentitation of fibroblasts into synthetic VSMCs.

**Conclusion:**

We characterized signatures of different cell types in normal and dissected human ascending aorta and identified a new marker for isolation of synthetic VSMCs. Moreover, we proposed a potential mechanism that synthetic VSMCs might interact with neutrophils and fibroblasts via CXCL12-CXCR4/ACKR3 axis whereby deteriorating the progression of ATAD, which might provide new insights to better understand the development and progression of ATAD.

**Supplementary Information:**

The online version contains supplementary material available at 10.1186/s10020-022-00584-4.

## Introduction

Acute thoracic aortic dissection (ATAD) is a fatal emergency with high mortality characterized by the tear of intima, followed by separation of intima and media as well as blood flow swarming into pseudo-lumen, whereby promoting the rupture of aorta (Nienaber et al. 2016). From 1995 to 2015, the age and sex-adjusted incidence of ATAD was about 4.4 per 100,000 person-years, a little bit increased than previous studies (Sen et al. 2021). Notably, patients with smoking and hypertension history showed higher prevalence of ATAD without discrepancy of sex. Though surgical aortic replacement and thoracic endovascular aortic repairment (TEVAR) are available to type A and type B ATAD, respectively, the perioperative complications exert enormous risks on patients, especially the elders and patients with underlying diseases (Hashimoto et al. 2022; Uchida et al. 2021). Thus, it is urgent to demonstrate the molecular and cellular mechanisms to provide new insights for clinical practice.

Aorta is composed by several cell types with heterogenous subpopulations including vascular smooth muscle cells (VSMCs), endothelial cells (ECs), fibroblasts (FBs) as well as infiltrated neutrophils and monocytes/macrophages (Weng et al. 2022; Amabili et al. 2019; Kim et al. 2017). However, the heterogeneity of subpopulations in different cell types is hardly studied. Phenotypic alteration between synthetic and contractile VSMCs is a key biological process in the maintenance of aortic homeostasis, but there are no specific surface markers to distinguish synthetic and contractile VSMCs whereby performing further studies (Zhang et al. 2020). Neutrophil infiltration is a pivotal pathological feature in ATAD, which might be recruited by adventitial-derived CXCL1/CSF3 whereby triggering ATAD via secretion of MMP9 (Anzai et al. 2015; Chai et al. 2022). Fibroblast is the major cellular component of adventitia and play roles in regulating extracellular matrix (ECM) organization, maintaining the integrity of tissues and immune response (Yun et al. 2005). But the roles of FBs in the development and progression of ATAD as well as the interaction among different cell types are less studied. Single-cell RNA-sequencing (scRNA-seq) is an emerging technique by identification of single cell RNA transcriptome, which could provide clues to explore the cellular heterogeneity, interaction network and cell differentiation trajectory in different tissues and better understand the pathogenesis of diseases from molecular and cellular level (Zhang et al. 2021; Stuart et al. 2019; Iinuma et al. 2022).

In this study, we performed scRNA-seq in human ascending aorta, analyzed the subpopulations of different cell types in normal and dissected ascending aorta, identified a specific surface marker for synthetic VSMCs and proposed potential interaction among VSMCs, neutrophils and FBs via CXCL12-CXCR4/ACKR3 axis. Overall, our study constructed gene expression landscape of different cell types in normal and dissected ascending aorta, which provided new insights to mechanisms in development and progression of ATAD.

## Methods

### Ethical statement and sample collection

The collection and use of human aortic samples were approved by the Ethical Committee of Shanghai Chest Hospital. Fresh ATAD (n = 5) and normal (n = 4) ascending aortic samples were obtained from patients with Stanford type A ATAD who have underwent surgical procedures and the healthy donors (Additional file 1: Fig. SIA) without cardiovascular diseases, respectively. All ATAD samples were obtained from intraoperative identified ascending aortic tissues of intimal tear (Additional file 1: Fig. SIB). These full-thickness samples with intimal tear, intimomedial flap and residual media-adventitia complex were stored in preserving buffer for scRNA-seq. Other normal and ATAD samples were divided into medial and adventitial tissues. In control group, 15 ascending aortic medial tissues and 11 adventitial tissues were collected. In ATAD group, 15 ascending aortic medial tissues and 11 adventitial tissues were collected. All medial samples in ATAD group used for IHC and IF were obtained from intimomedial flap with removement of intima. All adventitia samples in ATAD group used for IHC and IF were separated from residual media-adventitia complex near to intimal tear. All ATAD samples used for scRNA-seq, IHC and IF were washed with sterile PBS for several times to remove the residual blood and thrombus. Then these samples were fixed in 4% PFA for IHC and IF. Patients diagnosed with bicuspid aortic valve, Ehlers-Danlos syndrome, familial thoracic aortic aneurysm and dissection, chronic ATAD and acute Stanford Type A intramural hematoma were excluded from this study. All information of patients and donors were available. Patient demographics were shown in Additional file 1: File II.

### Statistical analysis

The processing of scRNA-seq data, methods to identify marker genes and differentially expressed genes for each cell type and subpopulation were shown in Additional file 1: Supplementary materials. Briefly, t-SNE and DEGs analysis were performed by Wilcox rank sum test algorithm following criteria including: lnFC > 0.25, p value < 0.05 and min.pct > 0.1. Significant mean and Cell Communication significance (p-value < 0.05) for cell communication were calculated based on the interaction and the normalized cell matrix achieved by Seurat Normalization. The WGCNA R package was used for WGCNA analysis, and Pearson correlations between module eigengene and different cell types were calculated.

## Results

### Cell subpopulation characteristics of human ascending aorta

Upon quality control and normalization, 39,525 cells were used for further analysis, and the population characteristics of 9 samples were shown in Additional file 1: File I. In this study, 14 clusters were primarily obtained (Additional file 1: Fig. SIA). Upon examination of conserved genes in each cluster, 8 cell types were identified, including VSMCs, ECs, 5 clusters of FBs, macrophages, 2 clusters of monocytes, T lymphocytes, mast cells and 2 clusters of neutrophils. The proportion of each cluster between control and ATAD group was shown in Additional file 1: Fig. SIB. The marker genes of these clusters were shown in Additional file 1: Fig. SIC-D.

In 3 non-immune cells, most VSMCs highly expressed CALD1, but the traditional contractile marker MYH11 (Milewicz et al. 2017) and synthetic marker MYH10 (Harrison et al. 2019; Wang et al. 2020a) exhibited a separated expression pattern, indicating the existence of contractile and synthetic phenotype of VSMCs. In those immune cells, a small proportion of neutrophils showed higher levels of S100A12 and CD177, implying activation of these cells. Though all monocytes highly expressed CD163, their markers exhibited a splitted trend, which characterized by high levels of SERPINB2 and EREG in monocytes 1 as well as overexpression of MT1G in monocytes 2. These results revealed the heterogeneity in each cell type and prompted us to explore the subpopulation composition of these cell types.

### Heterogeneous subpopulations of VSMCs in ascending aorta

**Fig. 1:**
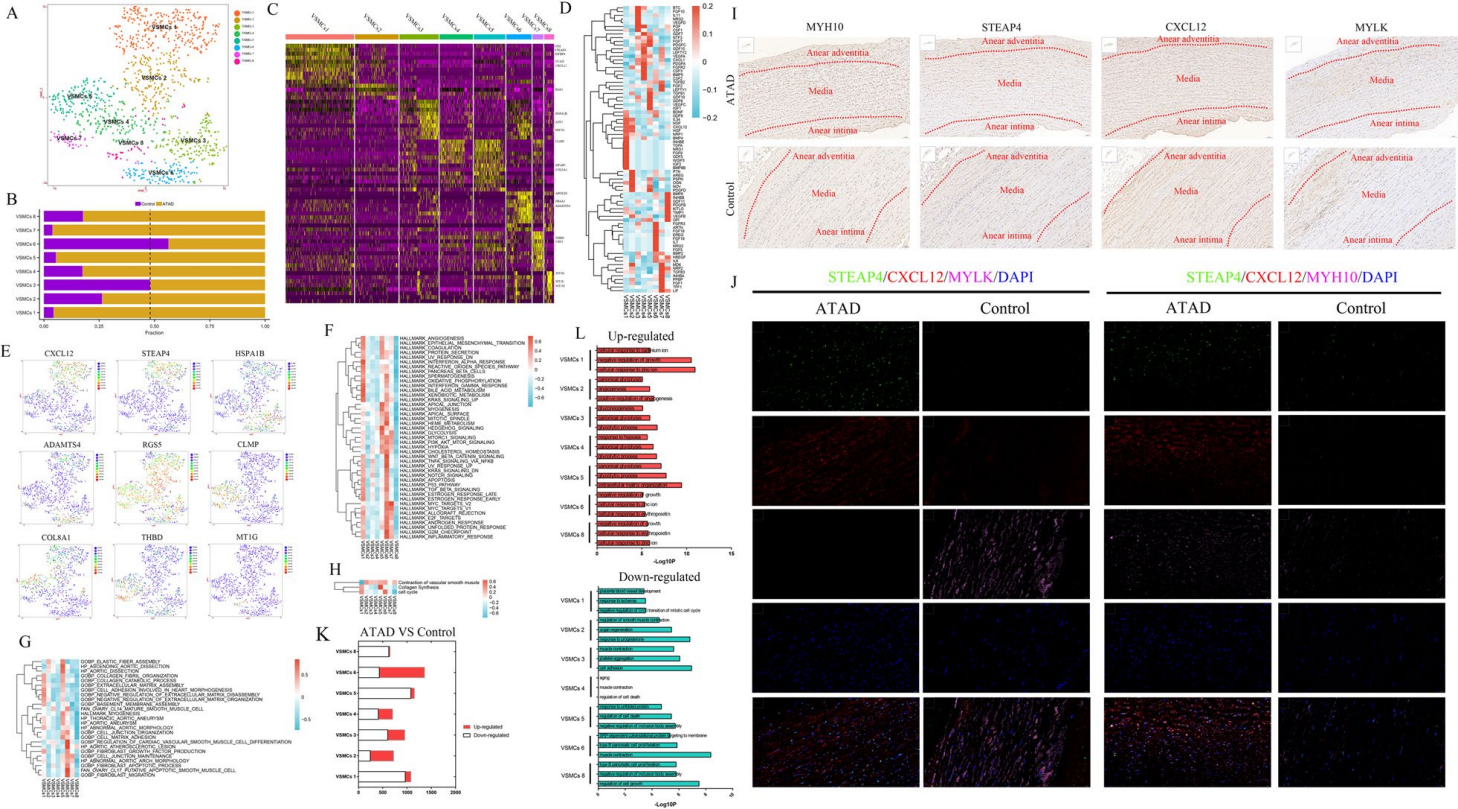
Eight subpopulations of VSMCs were identified with different functions and markers including 2 new markers for synthetic VSMCs. **A** t-SNE plot showed 8 subpopulations of VSMCs upon re-clustering. **B**the proportion of each subpopulation of VSMCs between control and ATAD. The dashed line was used to discriminate the dominant subpopulations in ATAD and control group. **C** the heatmap of marker genes for each subpopulation of VSMCs. **D** the expression of growth factors in each subpopulation of VSMCs. **E** t-SNE plots displayed the expression of representative marker genes for each subpopulation of VSMCs. **F** Qusage analysis of hallmark gene-sets enrichment for each subpopulation of VSMCs. **G** Qusage analysis of selected gene-sets enrichment relating to ATAD and functions of VSMCs for each subpopulation. **H** the results of Qusage analysis to identify functions of contraction, collagen synthesis and proliferation for each subpopulation of VSMCs. **I** IHC results showed higher expression of MYH10 (synthetic VSMCs markers), STEAP4 and CXCL12 in ATAD group, but the expression of MYLK (contractile VSMCs marker) was inverted. It also displayed similar expression characteristics of MYH10, STEAP4 and CXCL12 in same area except for MYLK. **J** IF revealed co-expression of STEAP4/CXCL12/MYH10 but not STEAP4/CXCL12/MYLK (contractile VSMCs marker) in control and ATAD group, with higher positive proportion of STEAP4/CXCL12/MYH10 in ATAD group. **K** differential expressed genes (DEGs) in subpopulations of VSMCs (ATAD/Control). **L** GO analysis for up-regulated and down-regulated genes in subpopulations of VSMCs (ATAD/Control)

We got 8 subpopulations upon re-clustering VSMCs (Fig. 1A). The composition of subpopulations in each sample was shown in Additional file 1: Fig. IIA. The proportion of each subpopulation in ATAD and control group was shown in Fig. 1B.

VSMCs 1 was identified as synthetic VSMCs for its higher expressions of complement activation, ECM and apoptotic genes including CXCL12, CFH, VCAN, MYH10 and IGFBP4 (Maridas et al. 2017), which also expressed growth factors such as BMP4, TGFA, NRG1, FGF9 and GDF5, whereby mediating cell–cell signaling, cell proliferation and differentiation (Fig. 1C–E, Additional file 1: Fig. IIB). The genes distinctly expressed in VSMCs 1 played roles in ECM and collagen metabolism, cell adhesion, antigen-processing and interferon response, which were consistent with the functions of synthetic VSMCs (Additional file 1: Fig. IIC–D). It also exhibited increased type I IFN response, ROS pathway and oxidative phosphorylation to regulate inflammation, oxidative stress and enhanced energy metabolism (Fig. 1F). Further analysis found its involvement in ECM modulation and moderate expression of collagen and cell cycle genes (Li et al. 2020) (Fig. 1G, H). Notably, we found specific expression of STEAP4 in VSMCs 1 (Fig. 1E), a protein mainly expressed on plasma membrane (Scarl et al. 2017). IHC and IF showed higher expressions of MYH10, STEAP4, CXCL12 in the same area of ATAD media, but the expression of contractile VSMCs marker MYLK was reversed. IF verified the expression of STEAP4 and CXCL12 in MYH10^+^, but not MYLK^+^ VSMCs of aortic media in both ATAD and control group (Fig. 1J, K).

We defined VSMCs 3 as stressed VSMCs for the expressions of HSPA1B, ATF3 and SOCS3 (Fig. 1C, E, Additional file 1: Fig. IIB). Genes uniquely expressed in VSMCs 3 modulated sarcomere organization, cardiac muscle cell apoptosis and signal transduction (Additional file 1: Fig. IIC, F). QuSage analysis revealed its moderate enrichment in vascular contraction and activation of TNF-α and notch signaling pathways (Fig. 1F–H).

VSMCs 6 might be proliferating VSMCs based on the expressions of cell proliferation and growth factor response genes such as APOLD1 (Basic et al. 2019), ADAMTS4 and NR4A3 (Hirano et al. 2019), with higher levels of growth factors including FGFR3, FGF18, FGF19, FGF5 and ARTN to regulate cell proliferation, differentiation and survival (Fig. 1C–E, Additional file 1: Fig. IIB). Its distinctly expressed genes regulated gene transcription and cell cycle (Additional file 1: IIC, IIH). We also found its activation of PI3K-Akt-mTOR, wnt-β-catenin, TNF-α, notch and inflammatory response signaling pathways, with the function of VSMCs differentiation and higher levels of cell cycle and VSMC contraction genes, which rendered the differentiation and proliferation activity of VSMCs 6 (Fig. 1F–H).

VSMCs 2, 4, 5 were identified as contractile VSMCs for their higher expression of RGS5 (Fig. 1C, E), a gene involved in arteriogenesis (Arnold et al. 2014). Notably, VSMCs 2 expressed growth factors including PTN, AREG, PSPN and OGN to improve cell survival, with distinct functions in cell death and actin filament capping (Fig. 1D, Additional file 1: Fig. IIC, IIF), which showed relatively higher enrichment of elastic fiber assembly and VSMCs contraction gene-sets (Fig. 1G, H). VSMCs 4 and 5 both expressed cell adhesion and apoptotic genes including CLMP (Werf et al. 2012) and EGLN3 (Li et al. 2019), with the expressions of growth factors such as GDFs, BMP5, FGFR2 and TGFB1 whereby modulating cell growth and development (Fig. 1C–E, Additional file 1: Fig. IIB). They also exhibited similar functions in glycolytic process, protein metabolism and apoptosis (Additional file 1: Figure IIC, IIG). Qusage analysis showed their enrichment of glycolysis and VSMCs contraction, implicating the alteration of energy metabolism in these subpopulations (Fig. 1F, H). Furthermore, VSMCs 5 showed relatively higher expressions of COL8A1 and MFAP5 (Fig. 1C, E, Additional file 1: IIB) as well as involvement in ATAD, activation of hedgehog signaling pathway and enhanced collagen synthesis (Fig. 1F–H).

VSMCs 7 was defined as monocyte-like VSMCs for its expressions of monocyte markers CD93 and THBD (Fig. 1C, E, Additional file 1: Fig. IIB), which lost the function of vascular contraction but showed enhanced gene transcription and glycolysis (Fig. 1F, H, Additional file 1: Fig. IIC, III). VSMCs 8 highly expressed metallothionein superfamily genes including MT1G and MT1M (Fig. 1C, E, Additional file 1: Fig. IIB), which distinctly regulated cell response to metal ion (Additional file 1: Fig. IIC, IIJ).

DEGs of VSMCs between ATAD and control group were shown in Fig. 1K. The results showed most subpopulations of VSMCs in ATAD group highly expressed genes involved in ECM organization, metal ion response, glycolysis and hypoxia, while exhibited lower levels of genes relating to cell adhesion and muscle contraction (Fig. 1L).

We noticed that most subpopulations of VSMCs in ATAD group exhibited higher proportions except for VSMCs 3 (stressed) and 6 (proliferating) (Fig. 1B). Though the augmentation of synthetic VSMCs in ATAD has been widely accepted, the higher proportion of contractile VSMCs was not consistent with previous studies.

### Differential phenotypes of FBs in ascending aorta

**Fig. 2:**
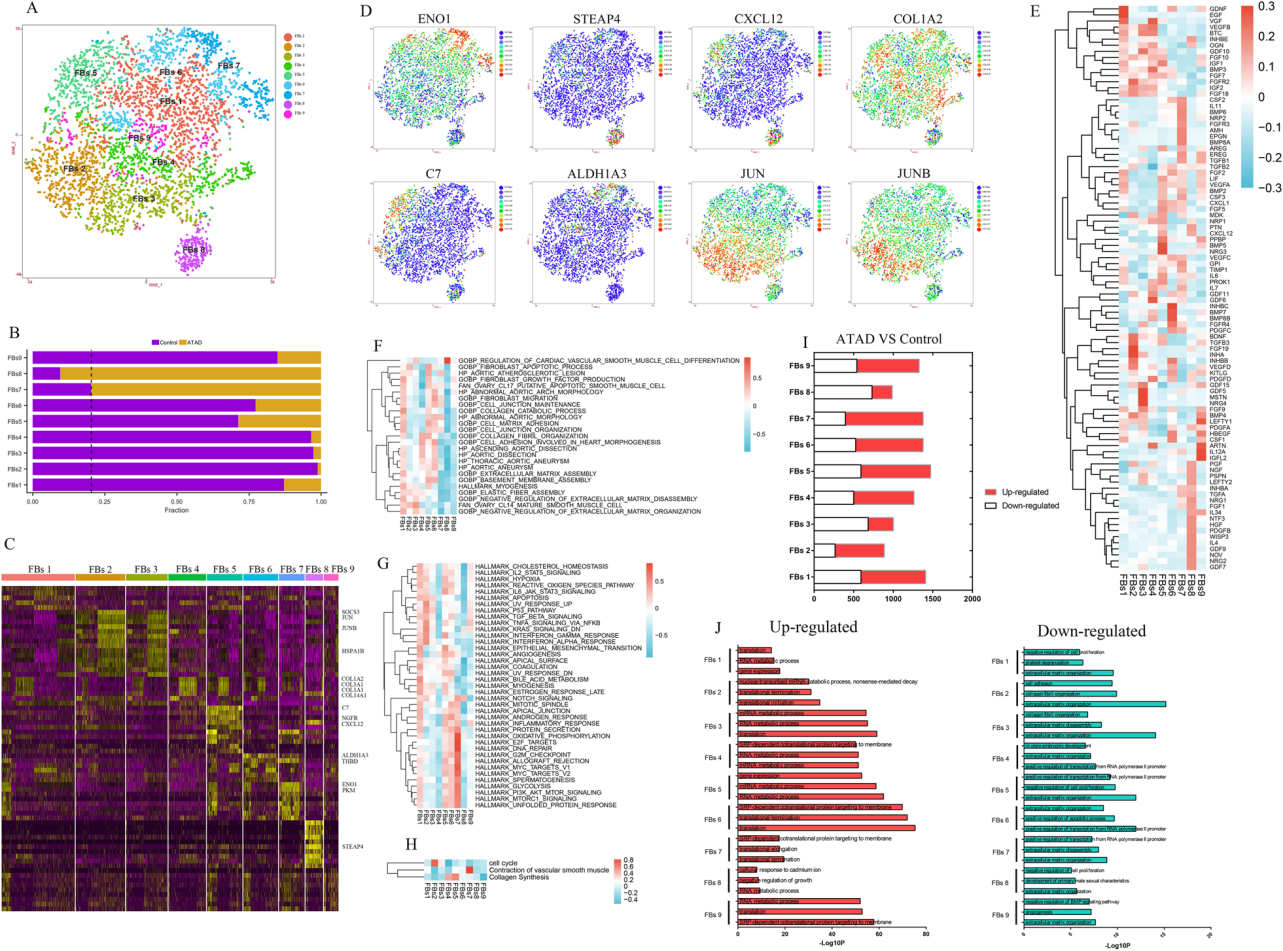
Nine subpopulations of FBs were identified with differential markers and functions. **A** t-SNE plot showed 9 subpopulations of FBs after re-clustering. **B** The proportion of each subpopulation of FBs between control and ATAD group. The dashed line was used to distinguish the dominant subpopulations in ATAD and control group. **C** Heatmap of marker genes for each subpopulation of FBs. **D** t-SNE plots showed the expressions of representative marker genes for each subpopulation of FBs. **E** The expressions of growth factors in subpopulations of FBs. **F** Qusage analysis of selected gene-sets enrichment relating to ATAD and FBs functions for each subpopulation of FBs. **G** Qusage analysis of hallmark gene-sets enrichment for each subpopulation of FBs. **H** the results of Qusage analysis to identify functions of contraction, collagen synthesis and proliferation for each subpopulation of FBs. **I** DEGs in subpopulations of FBs (ATAD/Control). **J** GO analysis for up-regulated and down-regulated genes in subpopulations of FBs (ATAD/Control)

Nine subpopulations were obtained after re-clustering 5 clusters of FBs (Fig. 2A). The composition of subpopulations in each sample and proportion of each subpopulation between ATAD and control group were shown in Additional file 1: Fig. IIIA and Fig. 2B, respectively.

FBs 7 highly expressed glycolytic genes including ENO1 (Wang et al. 2020b) and PKM as well as THBD, (Fig. 2C, D, Additional file 1: IIIB), with high levels of growth factors including EPGN, IL11 and NRP2 to support cell proliferation, migration and cardiovascular function (Fig. 2E). It also distinctly expressed genes relating to cellular component movement and angiogenesis (Additional file 1: Fig. IIIH). Qusage analysis demonstrated its functions in activation of PI3K-Akt-mTOR, DNA repair, oxidative phosphorylation and glycolysis, implicating the elevated requirement to energy (Fig. 2G). We also found its function in VSMC contraction and relevance to aortic atherosclerotic lesion (Fig. 2F, H).

FBs 8 was defined as synthetic VSMCs-like FBs for its higher expressions of STEAP4 and CXCL12, with elevated levels of growth factors containing GDF7, GDF9, TGFA and NRG1 whereby promoting cell proliferation and differentiation (Fig. 2C–E). Furthermore, it displayed unique functions in ECM organization, cell adhesion and blood vessel remodeling (Additional file 1: Fig. IIII). Qusage analysis revealed its roles in VSMCs differentiation and moderate activation of notch signaling pathway (Fig. 2F, G). These characteristics were in consistent with the functions of synthetic VSMCs, implying that FBs might differentiate into synthetic VSMCs.

FBs 1, 4, 5 and 6 were identified as collagen-synthetic FBs for their expressions of collagen genes including COL1A2, COL3A1, COL1A1 and COL14A1 (Fig. 2C, D, Additional file 1: Fig. IIIB). Though these FBs exhibited differential expressions of growth factors, most of them played roles in cell growth and survival (Fig. 2E). FBs 1 and 6 showed analogical functions in cell proliferation and migration, but FBs 4 exhibited functions in ECM organization, disassembly and collagen metabolic process (Additional file 1: Fig. IIIC, D, IIIF). Interestingly, FBs 5 showed enhanced level of immune-associated gene C7 and NGFR, an indicator of phenotype switching (Boshuizen et al. 2020) (Fig. 2C, D, Additional file 1: Fig. IIIB). It also distinctly regulated ribosome biogenesis (Additional file 1: Fig. IIIC, IIIG). Qusage analysis further revealed the activation of IL6-JAK-STAT3 signaling pathway and response to IFN-γ as well as enrichment of cell–matrix adhesion, collagen fibril organization and collagen synthesis for FBs 5 (Fig. 2F–H). These results hinted us FBs 5 might be involved in phenotype switching to maintain aortic homeostasis. FBs 6 specifically expressed ALDH1A3, a gene relating to various metabolic processes, cell proliferation and regulating the expression of ECM proteins (Xie et al. 2019) (Fig. 2C, D). Upon Qusage analysis, FBs 6 regulated cell migration, angiogenesis and TGF-β signaling, with moderate enrichment of cell adhesion, migration and relevance to abnormal aortic arch morphology, aortic dissection and aneurysm (Fig. 2F, G).

FBs 2 and 3 might be stressed FBs for their overexpressions of HSPA1B, SOCS3, JUN and JUNB (Fig. 2C, D, Additional file 1: Fig. IIIB). FBs 2 and 3 uniquely regulated transcription (Additional file 1: Fig. IIIC, IIIE). Qusage analysis revealed their roles in apoptosis and activation of TNF-α, IFN α and γ response signaling pathways (Fig. 2G).

Though FBs 9 expressed several collagen genes (Fig. 2C, D), it showed no specific markers. Moreover, it functioned in neutrophil chemotaxis and inflammatory response (Additional file 1: Fig. IIIC, IIIJ), which might be involved in the process of neutrophil infiltration.

DEGs of FBs between ATAD and control group were shown in Fig. 2I. The results showed most subpopulations of FBs in ATAD group highly expressed genes of transcriptional and translational processes, while exhibited lower levels of genes regulating collagen and ECM organization (Fig. 2J).

As the major cell type of adventitia, most FBs exhibited higher proportions in control group (Fig. 2B). Nevertheless, FBs 7 and 8 (synthetic VSMCs-like FBs) were 2 dominant subpopulations in ATAD group (Fig. 2B), which might be associated with the development of ATAD and prompt us to study their relationship with synthetic VSMCs.

### Unique subpopulations of ECs in ascending aorta

**Fig. 3:**
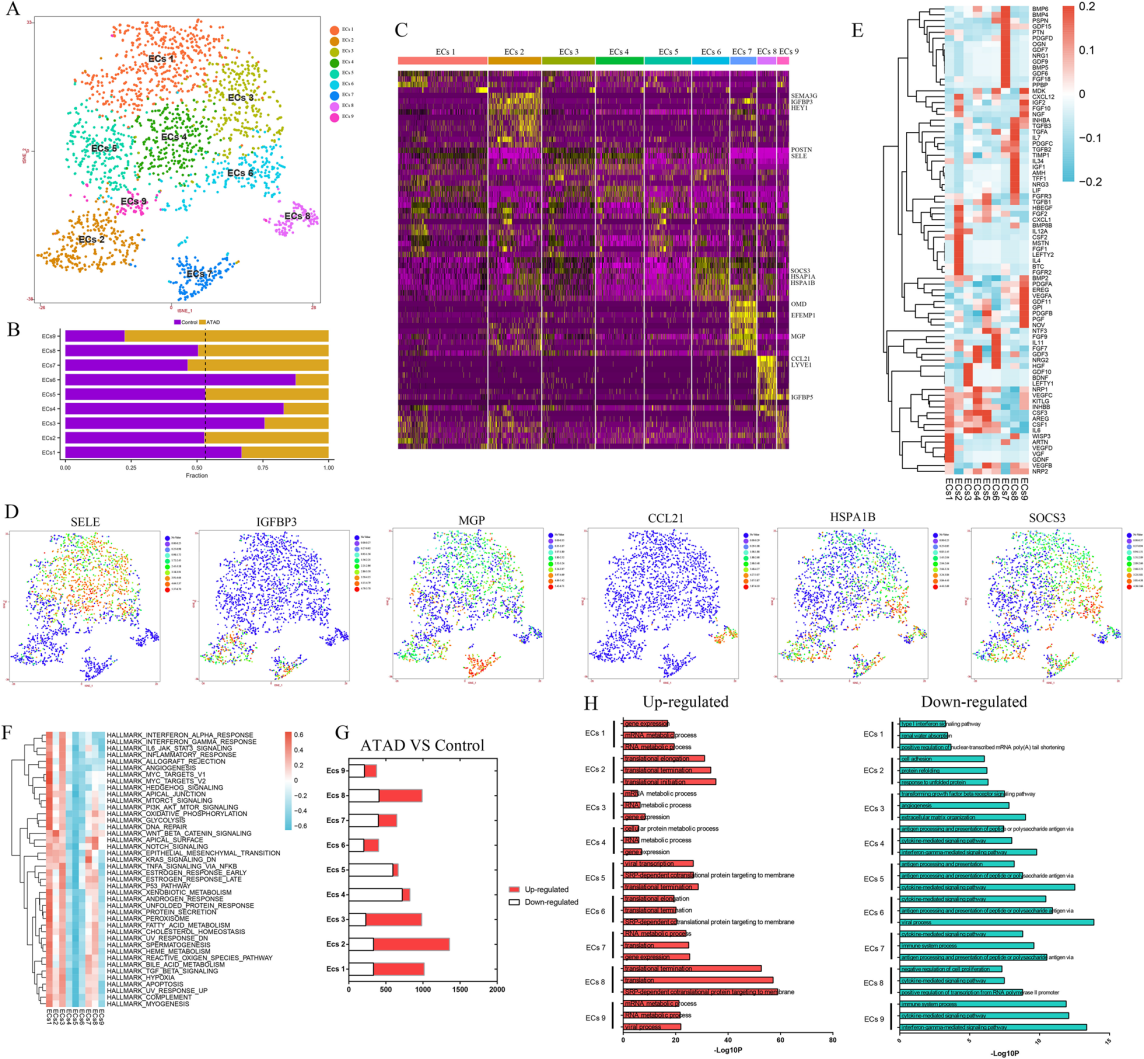
Nine subpopulations of ECs were obtained upon re-clustering with distinct markers and functions. **A** t-SNE plot showed 9 subpopulations of ECs. **B** The proportion of each subpopulation of ECs between control and ATAD group. The dashed line marked off the dominant subpopulations in ATAD and control group. **C** Heatmap of marker genes for each subpopulation of ECs. **D** t-SNE plots showed the expressions of representative marker genes for each subpopulation for ECs. **E** The expressions of growth factors for each subpopulation of ECs. **F** Qusage analysis of hallmark gene-sets enrichment for each subpopulation of ECs. **G** DEGs in subpopulations of ECs (ATAD/Control). **H** GO analysis for up-regulated and down-regulated genes in subpopulations of ECs (ATAD/Control)

ECs was re-clustered and identified 9 heterogenous subpopulations (Fig. 3A). The characteristics of populations in each sample and proportion of each population between ATAD and control group were shown in Additional file 1: Fig. IVA and Fig. 3B, respectively.

ECs 1, 3, 4 and 5 were identified as canonical ECs for their expressions of tissue development and vascular adhesion genes including POSTN and SELE (Fig. 3C, D, Additional file 1: Fig. IVB). ECs 1 and 3 highly expressed growth factors involved in endothelial growth and survival and angiogenesis such as ARTN, VEGFD, GDNF, HGF, GDF10 and BDNF (Fig. 3E). Moreover, ECs 4 showed higher levels of FGF7 and NRG2 whereby regulating wound healing and response to stimulus, while ECs 5 expressed PDGFB, NTF3 and IL6 to participate inflammation (Fig. 3E). Furthermore, ECs 1, 3 and 4 showed similar functions in IFN-γ and cytokine-mediated signaling pathways and defense response to virus (Additional file 1: Fig. IVC–D). Notably, ECs 3 distinctly regulated cell response to stimulus (Additional file 1: Fig. IVC, IVF). ECs 4 also modulated multiple immune responses (Additional file 1: Fig. IVC, IVG). Unexpectedly, ECs 5 did not display special function. Qusage analysis revealed significant activation of IL6-JAK-STAT3, hedgehog, PI3K-Akt-mTOR and TNF-α signaling pathways in ECs 1 and 3 (Fig. 3F) to regulate inflammation and immune response and angiogenesis.

ECs 2 was identified as angiogenic ECs for its expressions of ECs migration, vascular modulation and development genes including SEMA3G (Liu et al. 2020), IGFBP3 (Luo et al. 2020) and HEY1 (Kung-Chun Chiu et al. 2019), with expressions of growth factors including FGF1, FGF2, HBEGF and CXCL1 to support angiogenesis and neutrophil chemotaxis (Fig. 3C–E, Additional file 1: Fig. IVB). Further analysis unveiled its distinct functions in angiogenesis and vasculogenesis (Additional file 1: Fig. IVC, IVE). Qusage analysis displayed the activation of wnt-β-catenin signaling pathway (Fig. 3F), which might be favorable to cell migration and promoted angiogenesis.

We deduced ECs 7 might be remodeling ECs for its expressions of ECM organization and tissue remodeling genes including OMD, EFEMP1 (Wang et al. 2020c) and MGP, which also partly expressed IGFBP3, with ubiquitous expressions of tissue remodeling growth factors such as BMP4-6 and OGN (Fig. 3C–E, Additional file 1: Figure IVB). Moreover, ECs 7 functioned in oxidation–reduction process, IL-5, IL-12, IFN-γ production and cell differentiation (Additional file 1: Fig. IVC, IVH). Qusage analysis revealed its activation of ROS, TGF-β and K-Ras signaling pathways (Fig. 3F).

ECs 8 might be lymphatic-like ECs for its expressions of lymphatic formation and chemokine genes including CCL21, LYVE1 and IGFBP5, with higher expressions of growth factors such as TGFB3, TGFB1, TGFA and LIF to support lymphangiogenesis (Fig. 3C–E, Additional file 1: Fig. IVB). It also regulated deacetylation of several proteins, PI3K activity and lymphangiogenesis (Additional file 1: Fig. IVC, IVI). Moreover, ECs 8 showed higher activity in notch signaling pathway and multiple metabolic processes (Fig. 3F).

**Fig. 4:**
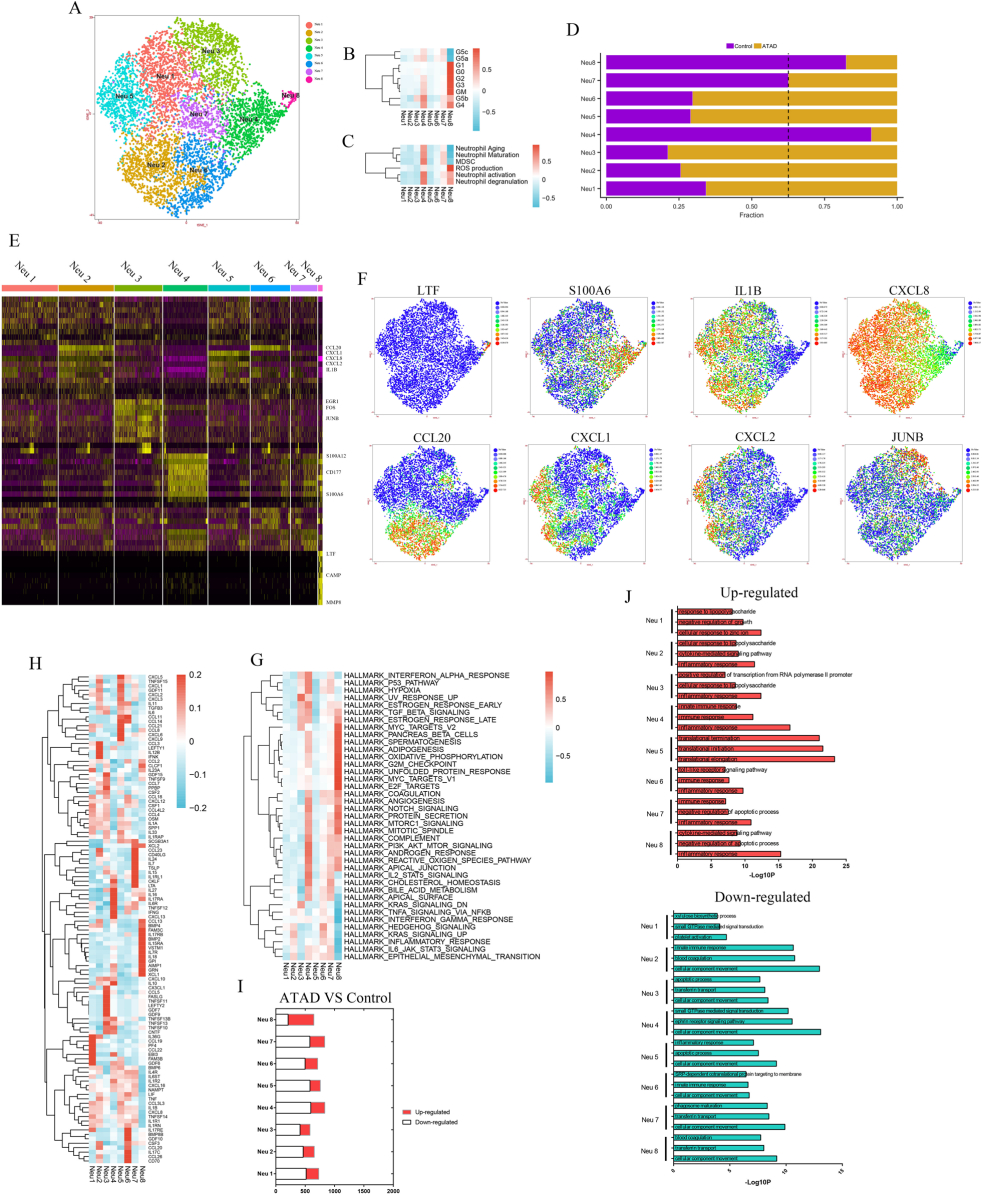
Eight subpopulations of neutrophils were identified with different markers, functions and stages. **A** t-SNE plot showed 8 subpopulations of neutrophils upon re-clustering. **B**, **C** Qusage analysis to identify the exact stage and function for each subpopulation of neutrophils. **D** the proportion of each subpopulation of neutrophils between control and ATAD group. The dashed line discriminated the dominant subpopulations in control and ATAD group. **E** heatmap of marker genes for each subpopulation of neutrophils. **F** t-SNE plots to show the expressions of representative marker genes for each subpopulation of neutrophils. **G** Qusage analysis of hallmark gene-sets enrichment for each subpopulation of neutrophils. **H** the expressions of cytokines for each subpopulation of neutrophils. **I** DEGs in subpopulations of neutrophils (ATAD/Control). **J** GO analysis for up-regulated and down-regulated genes in subpopulations of neutrophils (ATAD/Control)

ECs 6 exhibited higher levels of SOCS3, HSPA1A, HSPA1B, which regulated transcription, cell growth and death (Figs. 3C, D, 4G, Additional file 1: Fig. IVB–C, IVG). ECs 9 showed highly expressed lymphatic-like ECs marker IGFBP5 (Additional file 1: Fig. IVB), with activation of wnt-β-catenin signaling pathway (Fig. 3F). It also functioned in negative regulation of ECs migration and angiogenesis (Additional file 1: Fig. IVJ). These results indicated its potential origin from lymphatic-like ECs.

DEGs of ECs between ATAD and control group were shown in Fig. 3G. The results showed most subpopulations of ECs in ATAD group highly expressed genes involved in transcriptional and translational processes, while exhibited lower levels of genes relating to immune response, antigen processing and presentation (Fig. 3H).

### Subpopulations of infiltrated neutrophils in ascending aorta

Eight subpopulations were identified upon re-clustering neutrophils (Fig. 4A). For the differences between myeloid and peripheral neutrophils, we analyzed the markers and functions of neutrophils in ascending aorta according to the study conducted by Xie et al (2020). Neu 8 exhibited higher enrichment of G0, G1, G2, GM, G3 and G4 markers as well as functions in neutrophil activation, degranulation and ROS production, implying it might be a mixture of myeloid-derived neutrophils (Fig. 4B, C). On the contrary, Neu 4 and Neu 7 might be mature peripheral neutrophils for their higher enrichment of G4 and G5b markers as well as functions in neutrophil aging, maturation, activation and degranulation, but Neu 1–3, 5 and 6 showed no enrichment in these markers and functions (Fig. 4B, C). The composition of subpopulations in each sample was shown in Additional file 1: Fig. VA. The proportions of Neu 8, 4 and 7 exhibited higher levels in control group, but Neu 1–3, 5 and 6 were dominant subpopulations in ATAD group (Fig. 4D), implying Neu8, 4 and 7 might be the main subpopulations in physiological condition.

**Fig. 5:**
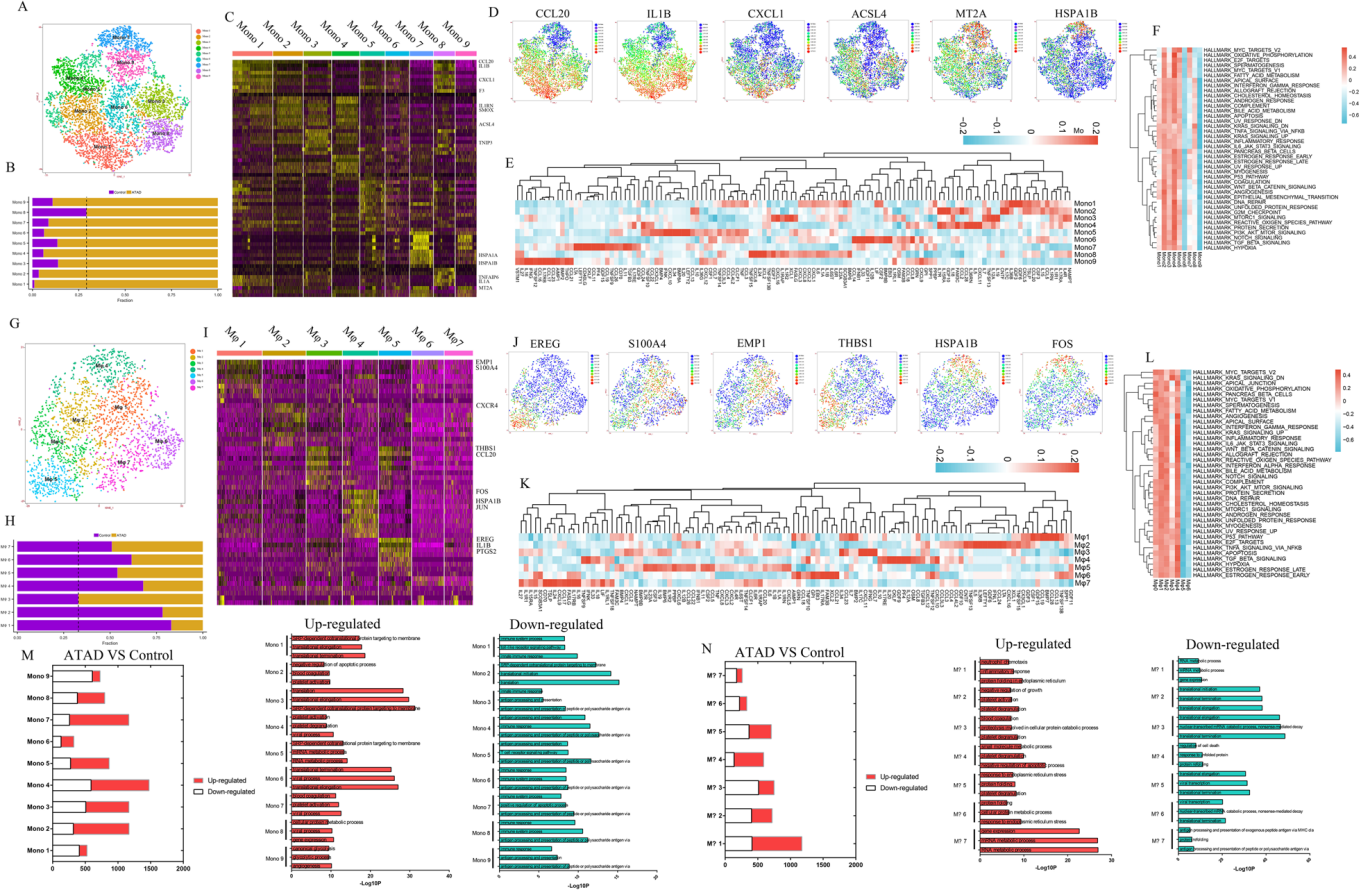
Nine subpopulations of monocytes and 7 subpopulations of macrophages were identified with different markers and functions. **A** t-SNE plot showed 9 subpopulations of monocytes upon re-clustering. **B** the proportion for each subpopulation of monocytes between control and ATAD group. The dashed line represented the highest proportion of Mono 8 in control group. **C** heatmap of marker genes for each subpopulation of monocytes. **D** t-SNE plots showed the expressions of representative marker genes for each subpopulation of monocytes. **E** Qusage analysis of hallmark gene-sets enrichment for subpopulations of monocytes. **F** the expressions of cytokines for subpopulations of monocytes. **G** t-SNE plot exhibited 7 subpopulations of macrophages. **H** The proportion for each subpopulation of macrophages between control and ATAD group. The dashed line was used to discriminate the dominant subpopulations in ATAD and control group. **I** Heatmap of marker genes for each subpopulation of macrophages. **J** t-SNE plots revealed the expressions of representative marker genes for each subpopulation of macrophages. **K** Qusage analysis of hallmark gene-sets enrichment for each subpopulation of macrophages. **L** the expressions of cytokines for subpopulations of macrophages. **M** DEGs and GO analysis for up-regulated and down-regulated genes in subpopulations of monocytes (ATAD/Control). **N** DEGs and GO analysis for up-regulated and down-regulated genes in subpopulations of macrophages (ATAD/Control)

As previously described, Neu 8 expressed G2 and G3 neutrophil markers including LTF and CAMP as well as G4 neutrophil marker MMP8, with high levels of cytokines including CCL13, GPI, IL18 and AIMP1 to chemoattract monocyte and lymphocyte and induce leukocyte migration, angiogenesis and inflammation (Figs. 4E, F, 5H, Additional file 1: Fig. VB). It also functioned in mitochondrial DNA replication and translation, indicating its enhanced proliferation activity (Additional file 1: Fig. VC, VL). Moreover, Neu 8 displayed enhanced activities in oxidative phosphorylation, notch and mTORC1 signaling pathways (Fig. 4G).

Neu 4 and 7 exhibited similar markers including calcium-dependent signal transduction, neutrophil activity regulation and transmigration genes S100A12, S100A6 and CD177 with lower levels for the latter (Fig. 4E, F, Additional file 1: Fig. VB). Neu 4 highly expressed cytokines including IL16, IL27, CXCL13 and IL6R to regulate multiple immune response, while Neu 7 showed higher levels of IL24, IL7, IL15 and CCL23 to regulate apoptosis and immune cell activity (Fig. 4H). Both of them exhibited similar functions in chemotaxis, glycolysis and innate immune response (Additional file 1: Fig. VC, VJ). Moreover, Neu 4 regulated endocytosis, T cell tolerance induction, cell migration and TLR signaling pathway, while Neu 7 functioned in platelet activation, immune response and PI3K activity (Additional file 1: Fig. VC, VH, VK). Qusage analysis unveiled enhanced activities in IFN-α response, hypoxia, ROS, PI3K-Akt-mTOR and IL2-STAT5 signaling pathways for Neu 4 and 7 with lower for the latter (Fig. 4G).

Neu 1, 2, 5 and 6 showed higher expressions of IL1B and CXCL8, and regulated cell death, lipid and protein metabolism (Fig. 4E, F, Additional file 1: Fig. VB–D). Notably, Neu 1 expressed cytokines such as CCL19 and CCL22 to chemoattract different immune cells, whereas Neu 2 showed higher levels of CCL3 and IL12B to regulate inflammation and NK cell activation (Fig. 4H). Neu 5 expressed more chemokines represented by CCL11, CCL14, CCL21, CCL8, CXCL6 and CXCL9, implying its chemotactic activities for other immune cells, but Neu 6 expressed several lymphocyte, basophil and eosinophil chemotactic cytokines including CCL20, CC26 and CD70 (Fig. 4H). Furthermore, Neu 1 distinctly functioned in differentiation and metabolism (Additional file 1: Fig. VC, VE). Neu 2 and 6 regulated macrophage activation, chronic inflammation and integrin signaling pathway (Additional file 1: Fig. VC, VF). Qusage analysis further showed the moderate enrichment of Neu 6 in coagulation, angiogenesis and hedgehog signaling pathway (Fig. 4G).

Neu 3 was identified as stressed neutrophils for it gained stress-related genes including EGR1, FOS and JUNB (Fig. 4E, F, Additional file 1: Fig. VB), which uniquely functioned in innate immune response, necroptosis, mRNA processing and type I IFN signaling pathway (Additional file 1: Fig. VG). Interestingly, it showed mild enrichment of markers in G5a and G5b neutrophils (Fig. 4B), indicating its potential derivation from mature neutrophils.

DEGs of neutrophil between ATAD and control group were shown in Fig. 4I. The results showed most subpopulations of neutrophil in ATAD group highly expressed genes relating to immune and inflammatory response, while exhibited lower levels of genes associated with cell migration and innate immune response (Fig. 4J).

### Monocytes/macrophages subpopulations in ascending aorta

Nine clusters of monocytes and 7 clusters of macrophages were identified upon re-clustering (Fig. 5A, G). The composition of subpopulations for monocytes and macrophages in each sample were shown in Additional file 1: Fig. VIA and Additional file 1: Fig. VIIA. All subpopulations of monocytes were dominant in ATAD group (Fig. 5B).

Most cells of Mono 1–6 and 8 highly expressed immune-related genes including CCL20, IL1B and IL1RN (Fig. 5C, D, Additional file 1: Fig. VIB). Moreover, Mono 1 and 8 showed higher levels of CXCL1, TNFAIP6 (Gu et al. 2021), IL1A and F3, with expressions of several cytokines represented by GDF3, GDF6 and IL36B for Mono 1 as well as XCL2, IL24 and CXCL6 for Mono8, indicating their roles in neutrophil chemotaxis, inflammatory response and apoptosis (Additional file 1: Fig. VIB, Fig. 5E). Qusage analysis unveiled significant activation for Mono 1 and mild activation for Mono 8 of TNF-α and IL-6-JAK-STAT3 signaling pathways (Fig. 5F). Mono 2–4 highly expressed TNIP3, ACSL4 and SMOX, with higher levels of cytokines including members of CCL, CXCL and interleukin family, playing roles in apoptosis, ferroptosis, inflammation and chemotaxis (Fig. 5C–E, Additional file 1: Fig. VIB). Qusage analysis revealed their enrichment in angiogenesis, coagulation, oxidative phosphorylation and PI3K-Akt-mTOR activation (Fig. 5F). Mono 5 and 6 showed no specific markers, but expressed members of interleukin and CCL family, while Mono 5 exhibited similar gene-sets activity with Mono 2–4 (Fig. 5E, F). Furthermore, Mono 1, 2 and 4 showed similar functions in cell migration, IFN-γ response and T cell activation (Additional file 1: Fig. VIC–D). Mono 3 and 8 played roles in protein modification, TLR signaling pathway and T cell activity, with distinct functions of mono 3 in apoptosis, coagulation and platelet activation (Additional file 1: Fig. VIC, VIE–F). Mono 5 also regulated protein modification and TLRs activity (Additional file 1: Fig. VIC, VIG). Though Mono 7 and 9 showed higher expression of MT2A, only Mono 7 highly expressed stress-related genes HSPA1A and HSPA1B with functions in response to stress and protein modification (Fig. 5C, D, Additional file 1: Fig. VIB-C, VIH), which both functioned in response to metal ion and mineral absorption (Additional file 1: Fig. VII).

**Fig. 6:**
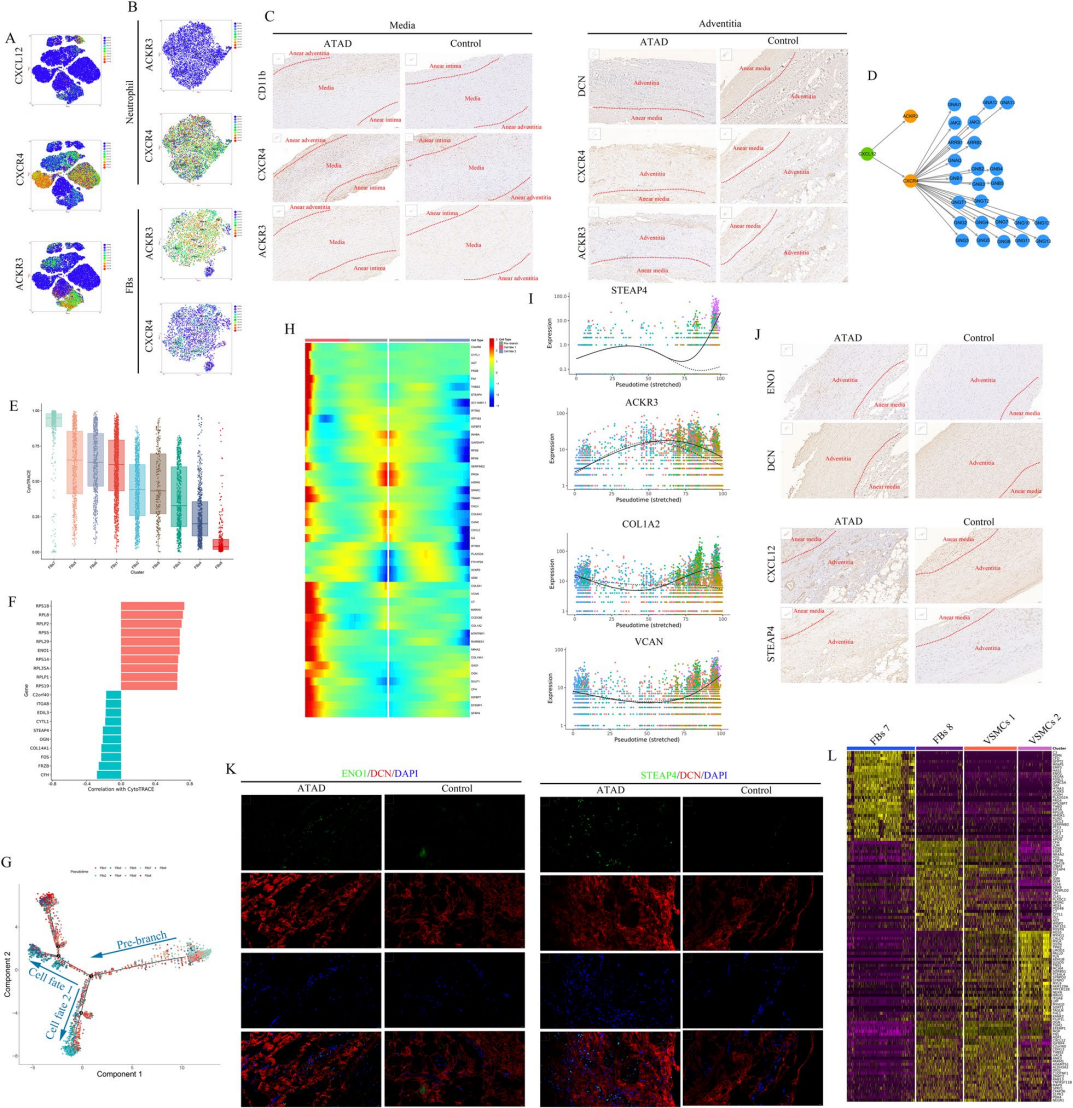
The interaction among VSMCs, FBs and neutrophils in control and ATAD group as well as cell differentiation trajectory of FBs. **A** t-SNE plots exhibited the expressions of CXCL12, CXCR4 and ACKR3 in all cell types. **B** t-SNE plots exhibited the expressions of CXCR4 and ACKR3 in neutrophils and FBs. **C** IHC revealed higher expression of CXCR4 in CD11b^+^ cells for media of ATAD group and ACKR3 in DCN^+^ cells for adventitia of ATAD group. **D** the predicted downregulation targets upon the combination of CXCL12 with CXCR4/ACKR3. **E** CytoTRACE predicted the cell differentiation potential of FBs. **F** genes correlated with more differentiated and less differentiated cells predicted by CytoTRACE. **G** pseudo-time analysis showed the cell differentiation trajectory of FBs upon selecting FBs 7 as the initiate. **H** the heatmap of gene expression alteration relating to ECM organization and cell proliferation. **I** the alteration curves of representative genes. The full line represented cell fate 1, the dashed line represented cell fate 2. **J** IHC showed higher expressions of ENO1 in DCN^+^ FBs from ATAD adventitia and higher proportion of CXCL12^+^/STEAP4^+^ cells in adventitia of ATAD group. **K** IF verified the higher expressions of ENO1 and STEAP4 in DCN^+^ FBs from ATAD adventitia. **L** heatmap showed similar expressions of marker genes between FBs 8 (synthetic VSMCs-like FBs) and VSMCs 1 (synthetic VSMCs) and distinct markers of FBs 7 and VSMCs 2

Mφ 1 and 3 were identified as monocyte-like macrophages for their differential expressions of monocyte markers. Mφ 1 expressed cell migration and apoptotic genes including S100A4 and EMP1, with high levels of several cytokines such as CCL13, CCL19 and CCL28 to recruit monocytes and lymphocytes, which regulated ECs function and protein modification (Fig. 5I–K, Additional file 1: Fig. VIIC-D). However, Mφ 3 expressed cell–cell adhesion and inflammation genes THBS1 and CCL20, with higher levels of CCL17, IFNG and CXCL11 to chemoattract lymphocytes, exerting effects on antigen processing and presentation as well as T cell proliferation and apoptosis (Fig. 5I–K, Additional file 1: Fig. VIIB–C, VIIE). Qusage analysis revealed similar activation of angiogenesis, IL6-JAK-STAT3 and PI3K-Akt-mTOR signaling pathways for Mφ 1 and 3 (Fig. 5L). We defined Mφ 2 as M2 macrophage for its higher expression of CXCR4 (Siefert et al. 2021) with expressions of several cytokines including XCL1, CXCL15 and IL5 to induce immune cell infiltration and inflammation, which regulated apoptosis, antigen processing and presentation, TLR and cytokine-mediated signaling pathways (Figs. 5I, 6K, Additional file 1: Fig. VIIB–C, VIIF). Qusage analysis displayed its similar enrichment to Mφ 1 and 3 (Fig. 5L). Mφ 4 was identified as stressed macrophage for its expressions of HSPA1B, FOS and JUN, which regulated transcriptional processes (Fig. 5I, J, Additional file 1: Fig. VIIB–C, VIIH). Mφ 5 might be M1 macrophage for its high levels of IL1B, PTGS2 and EREG, with the expressions of multiple cytokines including members of CCL, interleukin and CSF family, which also modulated apoptosis, adaptive immune response, inflammatory response, TLR and TNF signaling pathways (Fig. 5I–K, Additional file 1: Fig. VIIB–C, VIIG). Qusage analysis showed its significant enrichment in IFN-γ response, TNF-α and TGF-β and wnt-β-catenin signaling pathways (Fig. 5L). Mφ 6 and 7 did not showed specific markers and functions, which might under an unknown condition. Most subpopulations of macrophage were dominant in control group, while Mφ 3 exhibited higher proportion in ATAD group, implying its derivation from monocyte (Fig. 5H).

DEGs of monocytes and macrophages between ATAD and control group were shown in Fig. 5M, N. The results showed most subpopulations of monocytes in ATAD group highly expressed genes involved in transcriptional and translational processes, while exhibited lower levels of genes relating to immune response, antigen processing and presentation (Fig. 5M). Most sub-clusters of macrophages showed higher levels of genes regulating inflammatory response, coagulation and multiple metabolic processes, while exhibited lower levels of genes involved in transcriptional and translational processes and antigen processing and presentation (Fig. 5N).

### Synthetic VSMCs-derived CXCL12 mediated chemotaxis of neutrophils and transdifferentiation of FBs

Previous results demonstrated that VSMCs specifically expressed chemokine CXCL12, the ligand of CXCR4 and ACKR3, which propelled us to examine their expressions in different cell types. We found most FBs highly expressed ACKR3 except for FBs 8, and most neutrophils and T cells highly expressed CXCR4. Alternatively, ECs, monocytes and macrophages hardly expressed CXCL12, CXCR4 and ACKR3 (Fig. 6A, B). We hypothesized synthetic VSMCs-derived CXCL12 might exert regulatory effects on neutrophils, T cells and FBs. Upon re-clustering previously identified T cell, we first discriminated NK cell and T cell (Additional file 1: Fig. VIII). Then we re-clustered other T cells and identified 5 subpopulations including CD8-TEM, CD8-TEFF, CD4, naïve and stressed T cells with their specific markers (Additional file 1: Fig. IXA, IXC-D). However, all subpopulations of T cells in ATAD group exhibited lower or similar proportion compared with control group (Additional file 1: Fig. IXB). As acute lesion in ATAD, we predicted cell–cell communication when neutrophils and FBs were selected as the origin of receptor. Most subpopulations of neutrophils interacted with VSMCs 1 via CXCL12-CXCR4 in a mild intensity, while all subpopulations of FBs did not exhibit the interaction with VSMCs 1 via CXCL12-ACKR3 (Additional file 1: Fig. X, Additional file 1: Fig. XI). Nevertheless, IHC revealed the expressions of ACKR3 and CXCR4 in FBs of adventitia and neutrophils of media, respectively (Fig. 6C), implying the interaction between VSMCs 1 and FBs could not be excluded via CXCL12-ACKR3. The predicted downstream pathways of CXCL12-CXCR4 contained JAK-STAT, ERK1/2, PI3K-Akt and PLC-PKC signaling pathways and their potential targets (Fig. 6D), whereby regulating cytokine production, chemotaxis, ROS production, cell differentiation, migration and apoptosis. Unfortunately, related pathways and targets could not be predicted upon combination of CXCL12 with ACKR3.

To detect whether FBs 8 derived from other subpopulations of FBs, we predicted the differentiation trajectory of FBs via CytoTRACE and found FBs 7 was the initiate of cell differentiation trajectory, followed by collagen synthetic FBs 5, 6 and 1 (Fig. 6E). FBs 8, the synthetic VSMCs-like FBs, was the terminal state of FBs (Fig. 6E). Genes predicted to be correlated with less differentiated and more differentiated FBs were also screened. The genes involved in protein translation and elongation were associated with less differentiated FBs including RPS18, RPS5, RPL18 and RPL29 as well as ENO1 (Fig. 6F). However, synthetic VSMCs markers such as CFH and STEAP4 as well as bone development genes including FRZB and OGN showed higher correlation with terminal differentiated cells (Fig. 6F). These results implied the higher potential that ENO1^+^ FBs 7 might differentiate into other subpopulations. Pseudo-time analysis displayed 2 main branches in the cell differentiation trajectory upon selecting FBs 7 as the initiate, with the confluence of FBs 7 at initiate and FBs 8 at terminal (Fig. 6G, Additional file 1: Fig. IXE-F). FBs 1, 5 and 6 distributed all over the trajectory, but FBs 2, 3, 4 and 9 populated in 2 terminal branches (Additional file 1: Fig. IXE-F). Gene alteration along with the trajectory showed that synthetic VSMCs markers, ECM-related genes and genes correlated with more differentiated cells including STEAP4, CFH, VCAN, collagens, FRZB and OGN overexpressed after branching to cell fate 1, but the level of ACKR3 decreased at the terminal (Fig. 6H, I, Additional file 1: Fig. IXG, IXL). Nevertheless, stress-related and RNA catabolic genes such as FBLN2, HSPA1A, ATF3, EGR1 and HSPA6 overexpressed after branching to cell fate 2 (Additional file 1: Fig. IXH–K, IXM–N). IHC and IF revealed higher expression of ENO1 in DCN^+^ FBs in adventitia of ATAD group, moreover, IHC and IF also displayed higher proportion of CXCL12^+^/STEAP4^+^ cells and expression of STEAP4 in DCN^+^ FBs in adventitia of ATAD group (Fig. 6J–K). Homogeneity analysis among FBs 7, FBs 8, VSMCs 1 and VSMCs 2 also demonstrated similar marker genes between FBs 8 and VSMCs 1 (Fig. 6L).

### Cell differentiation trajectory of VSMCs and neutrophils

In consideration of the interaction among VSMCs, FBs and neutrophils, we further analyzed the cell differentiation trajectory of VSMCs and neutrophils.

**Fig. 7:**
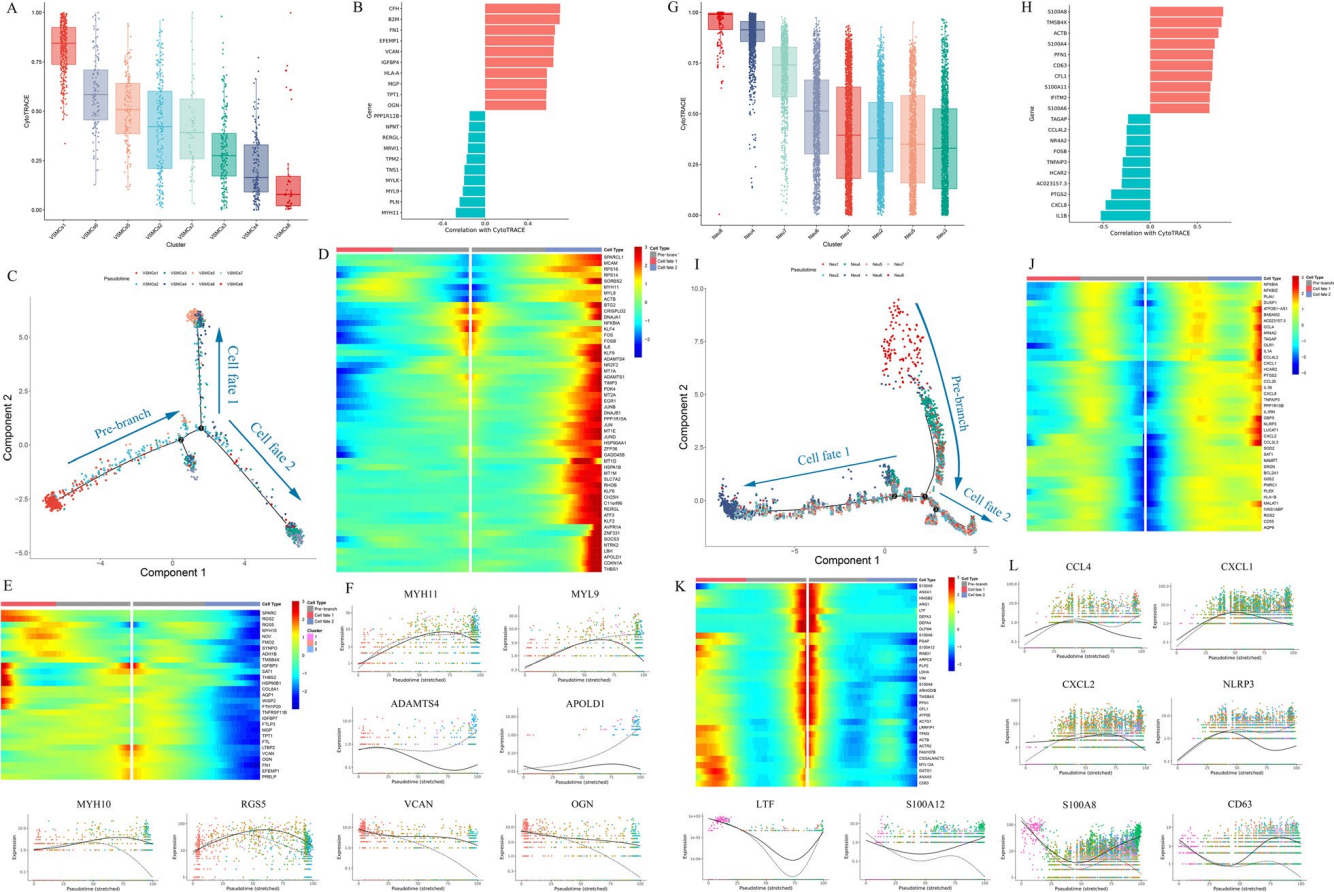
Cell differentiation trajectory and gene expression alteration of VSMCs and neutrophils. **A** CytoTRACE predicted the cell differentiation potential of VSMCs. **B** Genes correlated with more differentiated and less differentiated VSMCs predicted by CytoTRACE. **C** Pseudo-time analysis showed the cell differentiation trajectory of VSMCs based on selecting VSMCs 1 as the initiate. **D**, **E** heatmap of gene expression alteration relating to **D** ECM organization, cell adhesion and **E** response to stimulus. **F** Alteration curves of representative genes. The full line represented cell fate 1, the dashed line represented cell fate 2. **G** CytoTRACE predicted the cell differentiation potential of neutrophils. **H** Genes correlated with more differentiated and less differentiated neutrophils predicted by CytoTRACE. **I** pseudo-time analysis showed the cell differentiation trajectory of neutrophils upon selecting Neu 8 as the initiate. **J**, **K** heatmap of gene expression alteration relating to **J** chemotaxis, inflammatory and immune response and **K** cellular component movement. **L** Alteration curves of represented genes. The full line represented cell fate 1, the dashed line represented cell fate 2

CytoTRACE unveiled that VSMCs 1 was the initiate in the predicted trajectory with higher differentiation potential, followed by VSMCs 6, an intermediate state between synthetic VSMCs and contractile VSMCs, with subsequent 3 clusters of contractile VSMCs in order of VSMCs 5, 2, 4, and VSMCs 8 was the terminal state in differentiation trajectory with the loss of VSMCs markers (Fig. 7A). After filtration, we identified genes specifically correlated with less differentiated and more differentiated VSMCs. The expression of CFH, B2M, FN1, EFEMP1, VCAN and IGFBP4 showed more correlation with less differentiated VSMCs, while the expression of MYH11, PLN, MYL9, MYLK and TNS1 were more related with differentiated VSMCs (Fig. 7B). Upon VSMCs 1 was selected as the origin of cell differentiation, pseudo-time analysis of VSMCs exhibited 2 cell fates. VSMCs 2 resided all over of the trajectory, but VSMCs 3 and 4 populated in all branches except for the pre-branch. The terminal branches were populated by VSMCs 5 and 7 for cell fate 1 as well as part of VSMCs 6 and VSMCs 8 for cell fate 2 (Fig. 7C, Additional file 1: Fig. XIIA–B). After branching, the genes relating to metal ion, response to stimulus and contractile VSMCs markers overexpressed in cell fate 2 such as MYH11, MYL9, ADAMTS4, APOLD1, ATF3, MT1G and THBD (Fig. 7D, F, Additional file 1: Fig. XIID–E), but the expressions of synthetic VSMCs markers and ECM organization, cell adhesion and migration genes decreased in cell fate 2 including MYH10, RGS5, VCAN, VCAN, OGN and FRZB (Fig. 7E, F, Additional file 1: Fig. XIID, XIIF). Moreover, glycolysis, apoptosis and cell adhesion genes overexpressed in cell fate 1 represented by CLMP and EGLN3 (Additional file 1: Fig. XIIC-D, XIIG). This trajectory revealed the differentiation potential of VSMCs 1 and energy metabolism and function alteration of other subpopulations in cell development.

CytoTRACE analysis revealed Neu 8 was the initiate of differentiation trajectory with the highest differentiation potential, nearly followed by Neu 4 and 7, with other neutrophils in order of Neu 6-Neu 1-Neu 2-Neu 5 (Fig. 7G). The genes correlated with less differentiated neutrophils represented by S100A8, TMSB4X, S100A4, PFN1 and CD63, which modulated cell differentiation, proliferation and migration, while IL1B, CXCL8 and PTGS2 were significantly correlated with more differentiated neutrophils (Fig. 7H). Pseudo-time analysis revealed 2 terminal cell fates in this differentiation trajectory upon selecting Neu 8 as the initiate. Neu 8 and a fraction of Neu 4 were the only 2 subpopulations that existed in initial branch with higher differentiation potential, while most Neu 4 and other neutrophils distributed all over the trajectory with 2 different cell fates (Fig. 7I, Additional file 1: Fig. XIIH-I). Neutrophils located in cell fate 2 showed elevated expressions of genes relating to chemotaxis, inflammatory and immune response such as CCL4, CXCL1, CXCL2, CXCL8 and NLRP3 as well as decreased expression of Neu 8 marker LTF, indicating their roles in pro-inflammation (Fig. 7J, L, Additional file 1: XIIK–L). For cell fate 1, the genes of cellular component movement, immune response and Th1 cell activation exhibited high levels including S100A8, S100A12, S100A6, CD63, TNFRSF1B, IFITM3, IFITM2 and CST7 (Fig. 7K–L, Additional file 1: Fig. XIIJ–K, XIIM–N). These results demonstrated the differentiation potential of Neu8 and differential state of other subpopulations.

### Co-expression network among VSMCs, FBs and neutrophils

**Fig. 8:**
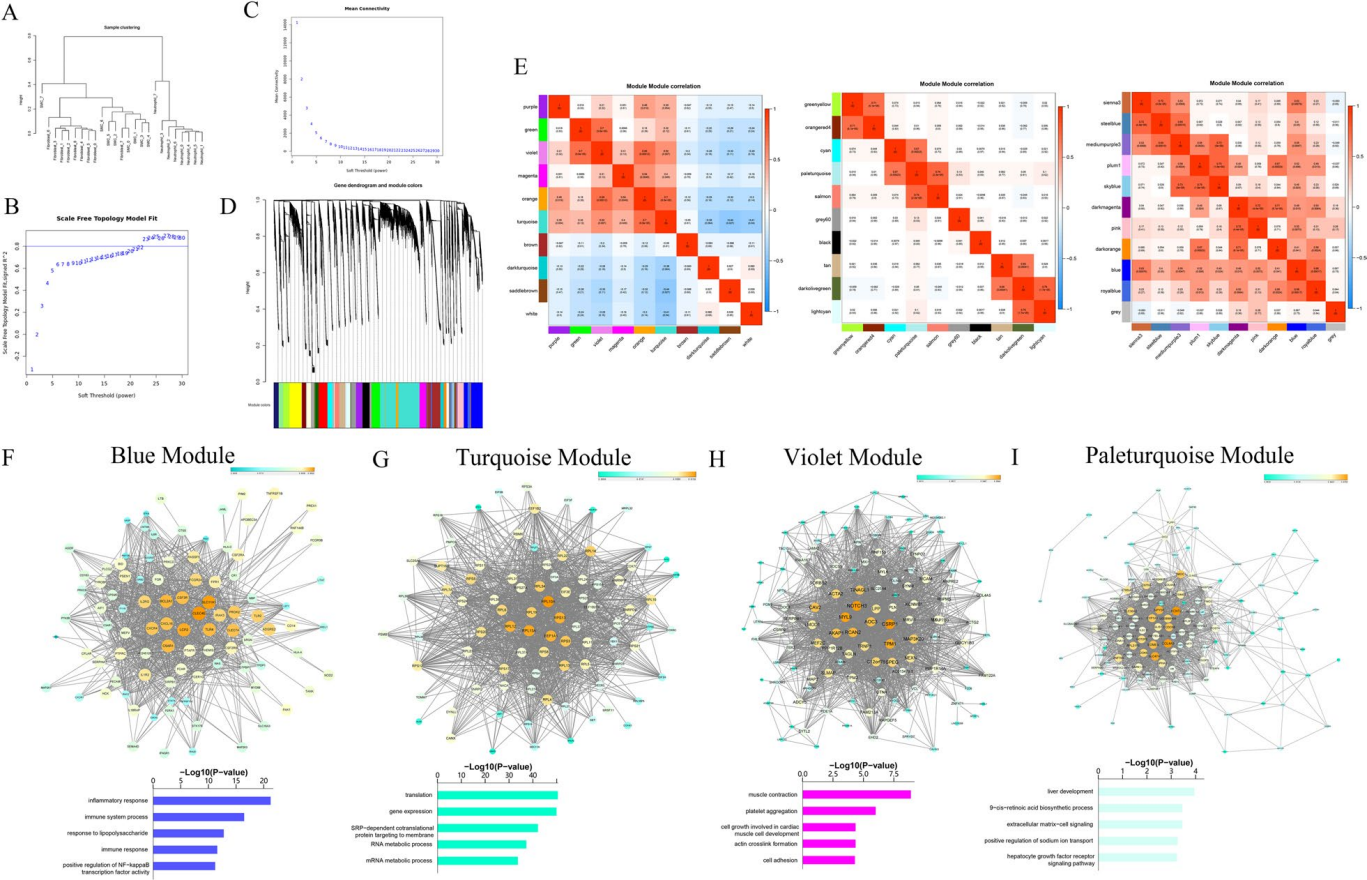
Correlated modules identified by WGCNA among VSMCs, FBs and neutrophils. **A** Sample cluster of VSMCs, FBs and neutrophils. **B**–**C** identification of soft threshold for WGCNA. **D**–**E** the hierarchical cluster dendrogram and correlation analysis identified 12 co-expression modules. **F**–**I** networks and gene functions for blue, turquoise, violet and paleturquoise module

WGCNA was performed to demonstrate the co-expression regulatory network among VSMCs, FBs and neutrophils (Fig. 8A–C). Twelve modules (Fig. 8D, E) were identified in regulation of different biological processes.

The distinctly correlated modules splitted neutrophils into 2 different parts including Neu 4, 7 and 8 characterized by expressions of genes in blue and royalblue module as well as Neu 2, 5 and 6 represented by expression of genes in sienna3, steelblue and mediumpurple module. Myeloid-derived and peripheral neutrophils including Neu 4, 7 and 8 highly expressed genes in blue and royalblue modules, which were characterized by involvement of innate immune response and inflammatory response (Fig. 8F, Additional file 1: Fig. XIIIA). The core genes such as SCL11A (Cunrath and Bumann 2019), CLEC4E, LCP2 (Wang and Peng 2021), SYK (Mocsai et al. 2010) and ITGAM in blue and royalblue module regulated susceptibility to the intracellular pathogens, TCR-mediated intracellular signal transduction, adherence of neutrophils, inflammatory and immune response.

Neu 2, 5 and 6 showed distinct expression of genes in highly correlated modules including mediumpurple3, sienna3 and steelblue, which played roles in cell–cell signal, adhesion and leukocyte migration (Additional file 1: Fig. XIIIB–D). Upon excluding untitled genes in these modules, we found the core regulators of these modules including RNU1-87P and LINC00676, which needed further studies to illustrate their functions.

All FBs and most VSMCs except for VSMCs 8 highly expressed genes in orange and turquoise module, which were responsible to cell adhesion, calcium-mediated signaling pathway, cGMP metabolism as well as translational and RNA metabolic process (Fig. 8G, Additional file 1: Fig. XIIIE). The hub genes of turquoise module including RPL10A, RPS13 and other members of RPL and RPS family, which regulated translation and RNA metabolic process. The core genes in orange module such as THRB (Liu et al. 2021), FRZB, MYH10 and FBLIM1 modulated growth, cell adhesion, cell morphology and cell motility. Moreover, VSMCs 2, 3, 4, 6 and 8 also distinctly expressed genes in green and violet module, which regulated muscle contraction, actin crosslink formation, cell adhesion and protein modification (Fig. 8H, Additional file 1: Fig. XIIIF). The key regulators of these modules including EIF2B3 (Lee et al. 2021), contractile VSMCs markers MYL9, TPM1 and TAGLN and NOTCH3 (Morris et al. 2019), participating in vascular development and VSMCs differentiation.

VSMCs 1 and FBs 8, with similar characteristics of marker genes, highly expressed genes in cyan, paleturquoise and salmon module, which functioned in ECM-cell signaling, cell adhesion, CCL2 secretion and multiple metabolic processes (Fig. 8I, Additional file 1: Fig. XIIIG–H). Upon filtration of untitled genes, PKP1 (Lee et al. 2017), DCHS2 and COL4A3 were identified as hub genes for these modules, acting as regulators of cell adhesion and ECM organization, which were in accordance with the functions of synthetic VSMCs.

### Immediate early genes (IEGs) in subpopulations of different cell types

Tissue dissociation induces expressions of IEGs and influences the accuracy in identification of cell subpopulations. We analyzed the expressions of dissociation-induced IEGs, and found most stressed subpopulations in different cell types conservatively expressed stress-related genes including HSPA1B, SOCS3 and JUN. Upon correlation analysis among all subpopulations on the basis of top 2000 variable genes, overall expression of dissociation-induced IEGs (Brink et al. 2017) for each subpopulation was calculated (Additional file 1: Fig. XIVA). FBs 2, FBs 3, FBs 9 and Mφ 4 exhibited higher overall expression of dissociation-induced IEGs, implying these subpopulations were influenced by tissue dissociation, which further intervened the identification of functions for these subpopulations (Additional file 1: Fig. XIVB).

## Discussion

IN this study, we analyzed subpopulations of different cell types existed in normal and dissected ascending aorta. Particularly, we identified STEAP4 as a new surface marker for synthetic VSMCs. Furthermore, we proposed a new insight that synthetic VSMCs-derived CXCL12 might recruit neutrophils and induce FBs to differentiate into synthetic VSMCs whereby deteriorating the progression of ATAD.

Though previous studies reported reduction of normal VSMCs and augmentation of apoptotic VSMCs were often seen in ATAD (An et al. 2017), more VSMCs were obtained from ATAD group but not control group. We speculated that the fragmentation of elastin and other ECM compositions caused by ATAD provided us an opportunity to obtain more VSMCs from these samples than normal samples with tight and well-organized ECM compositions. Stressed subpopulations existed in all cell types identified in our study. To get single-cell suspension, all samples were digested in collagenase I for 60 min to alleviate dissociation-induced stress. Nevertheless, FBs 2, FBs 3, FBs 9 and Mφ 4 exhibited higher overall expression of dissociation-induced IEGs, which led to biased identification of functions for these subpopulations.

Most subpopulations of different cell types in ATAD group exhibited aberrant expression of genes involved in transcriptional and translational processes as well as immune and inflammatory response, which might be favorable to the progression of ATAD.

STEAP4 is a gene expressed in both cytoplasm and membrane, which is reported to be a metalloreductase and participate in adipocyte development and chronic inflammation response (Zhao et al. 2022). We found STEAP4 specifically expressed in VSMC 1, the subpopulation speculated to be synthetic VSMCs, exhibiting relative higher specificity for synthetic VSMCs compared with MYH10, which might be a new marker for isolation of synthetic VSMCs. Though Li et al. reported the existence of a non-immune inflammatory cluster in ascending aorta which highly expressed macrophage markers C1QA and C1QB (Li et al. 2020), we did not find the expression of these 2 genes in non-immune cells including VSMCs, ECs and FBs (Additional file 1: Fig. XV). This difference might be the results of the distinctions between aortic aneurysm and ATAD.

Neutrophil is one of the most important immune cells infiltrated in aorta of ATAD, which involved in atherosclerosis, heart failure and myocardial infarction (Silvestre-Roig et al. 2020). Vafadarnejad et al. reported that neutrophils underwent the aging process from early stage to end stage in myocardial infarction and characterized by the enhanced expression of CXCR4 and diminished expressions of CD177 and MMP8 (Vafadarnejad et al. 2020). Here we unveiled augmented aging neutrophils with abnormal functions and decreased peripheral and myeloid-derived neutrophils in ATAD. Moreover, we found that Neu 8 was a mixture of G0, G1, G2, GM, G3 and G4 neutrophils reported by Xie et al., but Neu 4 and 7 mainly exhibited markers and functions of G5b (Xie et al. 2020), representing major subpopulations in physiological condition. In addition, other subpopulations of neutrophils were the dominance in ATAD group, which secreted more chemokines to attract immune cells and led to exasperated inflammation in ascending aorta.

FBs is the main cellular component in adventitia of aorta. Previous studies reported adventitial FBs-derived MCP-1 and KLF6 were favorable to recruitment of macrophage to promote the inflammatory response in dissected aorta (Thomson et al. 2020; Tieu et al. 2009; Liu et al. 2012). But the phenotypic characteristics of adventitial FBs remains less studied. We found FBs 8 shared similar markers, functions and hub genes with VSMCs 1, indicating its homogeneity to synthetic VSMCs. After prediction of cell differentiation trajectory via CytoTRACE, we identified FBs 7 exhibited relatively higher differentiation potential in FBs with higher expression of ENO1. These results implied that FBs in adventitia might differentiate into synthetic VSMCs in ATAD and provided us an opportunity to study the functions of FBs with higher differentiation potential. Unfortunately, we were unable to clarify whether FBs 8 derived from FBs 7 or other subpopulations of FBs, which will be investigated in our further studies.

CXCL12 was another specific marker for synthetic VSMCs revealed by our study, which is also known as SDF1, a chemokine with 2 receptors including CXCR4 and ACKR 3 and mainly expresses in FBs, stromal cells and epithelial cells (Chai et al. 2022). As a highly conserved 7 transmembrane regions protein, CXCL12 is the only ligand for CXCR4, which induces the activation of PI3K-Akt signaling pathway and regulates the phosphorylation of ERK1/2 to activate NF-κB and mTOR signaling pathways, thereby regulating cell growth and proliferation (Wu et al. 2010). Currently, we found ubiquitous expression of CXCR4, which might be recruited to aorta by CXCL12 signaling from VSMCs 1 in ATAD and promote the progression of inflammation. However, the combination of CXCL12 with ACKR3 activates MAPK/ERK signaling pathway to regulate cell survival, migration and differentiation (Basic et al. 2019; Huynh et al. 2020). Unexpectedly, the cell–cell interaction via CXCL12-ACKR3 was not identified for lower regulatory intensity between VSMCs 1 and FBs limited by the CellPhone database (Additional file 1: File III), but IHC demonstrated ACKR3 expressed in FBs of adventitia. Hence, we cannot ignore the interaction between FBs and VSMCs to mediate transdifferentiation from FBs to synthetic VSMCs, which will be clarified in our further studies. Unfortunately, for the lack of fresh ATAD and normal ascending aortic samples to isolate synthetic VSMCs and FBs, we were unable to verify the regulatory mechanism of synthetic VSMCs-derived CXCL12 on neutrophils and FBs.

## Conclusion

IN conclusion, this study revealed the heterogenous subpopulations of different cell types in normal and dissected ascending aorta and identified STEAP4 as a new surface marker for synthetic VSMCs. Furthermore, we proposed VSMCs-derived CXCL12 might be a potential signaling to induce neutrophil activation and FBs differentiation into synthetic VSMCs in ATAD. These findings might provide new markers and insights to isolate synthetic VSMCs and better understand mechanisms leading to ATAD.

## Supplementary Information

**Additional file 1**. **Supplementary Figure I**. **Eight cell types in aortic tissues from control and ATAD group revealed by scRNA-seq**. A, intraoperative identified ATAD and resected ascending aortic tissues. B, After washing by sterile PBS to remove residual blood and thrombus, ATAD samples were stored in preserving buffer for scRNA-seq. C, t-SNE plot exhibited all 14 clusters and 8 cell types identified in this study. D, the proportion of each cell type in control and ATAD group. The dashed line showed the boundary to discriminate the dominance of each cell type in control or ATAD group. E, the heatmap of marker genes for each cluster. F, t-SNE plots to show the expressions of representative marker genes for each cell type. **Supplementary Figure II**. **The composition of VSMCs subpopulations in each sample and characteristics for each cluster of VSMCs**. A, integrative t-SNE plot displayed the composition of VSMCs subpopulations for each sample. VSMCs was not identified from ATAD 5. B-C, t-SNE and violin plots showed the expressions of other representative marker genes. D, heatmap to identify functional modules of genes distinctly expressed in subpopulations of VSMCs. E-J, GO analysis for genes distinctly expressed in subpopulations of VSMCs that was not shown in Fig.2. **Supplementary Figure III**. **The composition of FBs subpopulations in each sample and features for each cluster of FBs**. A, integrative t-SNE plot showed the composition of each subpopulation of FBs in each sample. B-C, t-SNE and violin plots revealed the expression of other representative marker genes for subpopulations of FBs. D, heatmap to identify functional modules of genes distinctly expressed in subpopulations of FBs. E-F, GO analysis for genes distinctly expressed in subpopulations of FBs that was not shown in Fig.3. **Supplementary Figure IV**. **The composition of ECs subpopulations in each sample and characteristics for each cluster of ECs**. A, integrative t-SNE plot showed the composition of each subpopulation of ECs in each sample. B, t-SNE and violin plots revealed the expression of other representative marker genes for subpopulations of ECs. C, heatmap to identify functional modules of genes distinctly expressed in subpopulations of ECs. D-I, GO analysis for genes distinctly expressed in subpopulations of ECs that was not shown in Fig.4. **Supplementary Figure V**. **The composition of neutrophils subpopulations in each sample and characteristics for each cluster of neutrophils**. A, integrative t-SNE plot showed the composition of each subpopulation of neutrophils in all samples. B, t-SNE and violin plots revealed the expressions of other representative marker genes for subpopulations of neutrophils. C, heatmap to identify functional modules of genes distinctly expressed in subpopulations of neutrophils. D-I, GO analysis for genes distinctly expressed in subpopulations of neutrophils that was not shown in Fig.5. **Supplementary Figure VI**. **The composition of monocytes subpopulations in each sample and characteristics for each cluster of monocytes**. A, integrative t-SNE plot showed the composition of each subpopulation of monocytes in all samples. B, t-SNE and violin plots revealed the expressions of other representative marker genes for subpopulations of monocytes. C, heatmap to identify functional modules of genes distinctly expressed in subpopulations of monocytes. D-J, GO analysis for genes distinctly expressed in subpopulations of monocytes. **Supplementary Figure VII**. **The composition of macrophages subpopulations in each sample and characteristics for each cluster of macrophages**. A, integrative t-SNE plot showed the composition of each subpopulation of macrophages in all samples. B, t-SNE and violin plots revealed the expressions of other representative marker genes for subpopulations of macrophages. C, heatmap to identify functional modules of genes distinctly expressed in subpopulations of macrophages. D-H, GO analysis for genes distinctly expressed in subpopulations of macrophages. **Supplementary Figure VIII**. **Identification of T cell and NK cell as well as their expressions of marker genes**. **Supplementary Figure IX**. **The composition of T cells in control and ATAD group as well as cell differentiation trajectory and gene expression alteration of FBs among the differentiation trajectory**. A, t-SNE plot showed 5 subpopulations of T cells upon re-clustering. B, the proportion of each subpopulation in T cells. The dashed line discriminated the dominance of each subpopulation in control and ATAD group. C, heatmap of marker genes for each subpopulation of T cells. D, t-SNE and violin plots showed the expressions of representative marker genes for each subpopulation of T cells. E, the separated differentiation trajectory of FBs. F, the distribution of each subpopulation for FBs among the differentiation trajectory. G, alteration curves of other genes that was not shown in Fig.7. The full line represented cell fate 1, the dashed line represented cell fate 2. H-I, heatmap and curves of gene expression alteration relating to mRNA and translational processes. The full line represented cell fate 1, the dashed line represented cell fate 2. J-K, heatmap and curves of gene expression alteration relating to cell death and protein modification. The full line represented cell fate 1, the dashed line represented cell fate 2. **Supplementary Figure X**. **These bubble plots showed the interactions between VSMCs and subpopulations of FBs when FBs were selected as the origins of receptors**. **Supplementary Figure XI**. **These bubble plots showed the interactions between VSMCs and subpopulations of neutrophils when they were selected as the origins of receptors**. **Supplementary Figure XII**. **The cell differentiation trajectory and gene expression alteration of VSMCs and neutrophils**. A , the separated cell differentiation trajectory for each subpopulation of VSMCs. B, the distribution for each subpopulation of VSMCs among the differentiation trajectory. C, heatmap of gene expression alteration relating to glycolysis in subpopulations of VSMCs. D, alteration curves of other genes in subpopulations of VSMCs. The full line represented cell fate 1, the dashed line represented cell fate 2. E-G, GO analysis for 3 clusters of altered genes in subpopulations of FBs. H, the separated cell differentiation trajectory for each subpopulation of neutrophils. I, the distribution for each subpopulation of neutrophils among the differentiation trajectory. J, heatmap of gene expression alteration relating to immune response and Th1 cell activation in subpopulations of neutrophils. K, alteration curves of other genes in subpopulations of neutrophils. The full line represented cell fate 1, the dashed line represented cell fate 2. L-N, GO analysis for 3 clusters of altered genes in subpopulations of neutrophils. **Supplementary Figure XIII**. **The networks and functions for gene modules identified by WGCNA among neutrophils, VSMCs and FBs**. A-H, the networks and functions for gene modules identified by WGCNA that were not shown in Fig.8. **Supplementary Figure XIV**. **Overall expression of dissociation-induced IEGs in each subpopulation**. A, correlation analysis among all subpopulations on the basis of top 2000 variable genes. B, overall expression of dissociation-induced IEGs in each subpopulation. The red label represented positive expression of dissociation-induced IEGs. The green label represented positive expression of dissociation-induced IEGs. **Supplementary Figure XV**. **Expressions of C1QA and C1QB in non-immune cells including VSMCs, FBs and ECs**. **Supplementary file I**. **Separated cell cluster and marker gene of each cell type for all samples in this study**.** Supplementary file II**. **Patient demographics that were performed scRNA-seq**. **Supplementary file III**. **Genes that were used to identify functions of VSMCs and FBs markers of neutrophils**. **Supplementary file IV**. **The regulatory intensity results of CXCL12-ACKR3 between VSMCs 1 and FBs based on CellPhone database**. **Supplementary file V**. **The gene list of dissociation-induced IEGs**. **Supplementary file VI**. **The list of antibodies used in IHC and IF**.

## Data Availability

All the data in this study are available from the corresponding author for reasonable requests.

## Abbreviations

ATAD: Acute thoracic aortic dissection
TEVAR: Thoracic endovascular aortic repairment
VSMCs: Vascular smooth muscle cells
ECs: Endothelial cells
FBs: Fibroblasts
CXCL1: C-X-C chemokine ligand 1
CSF3: Colony stimulating factor 3
ECM: Extracellular matrix
CXCL12: C-X-C chemokine ligand 12
CXCR4: C-X-C chemokine receptor 4
ACKR3: Atypical chemokine receptor 3
PFA: Paraformaldehyde
CALD1: Caldesmon 1
MYH11: Myosin heavy chain 11
MYH10: Myosin heavy chain 10
S100A12: S100 calcium binding protein A12
SERPINB2: Serpin family B member 2
EREG: Epiregulin
MT1G: Metallothionein 1G
CFH: Complement factor H
VCAN: Versican
BMP4: Bone morphogenic protein 4
TGFA: Transforming growth factor-α
NRG1: Neuregulin 1
FGF9: Fibroblast growth factor 9
GDF5: Growth differentiation factor 5
IFN: Interferon
STEAP4: STEAP4 metalloreductase
MYLK: Myosin light chain kinase
HSPA1B: Heat shock protein family A (Hsp70) member 1B
SOCS3: Suppressor of cytokine signaling 3
APOLD1: Apolipoprotein L domain containing 1
ADAMTS4: ADAM metallopeptidase with thrombospondin type 1 motif 4
NR4A3: Nuclear receptor subfamily 4 group A member 3
FGFR3: Fibroblast growth factor receptor 3
ARTN: Artemin
PTN: Pleiotrophin
AREG: Amphiregulin
PSPN: Persephin
OGN: Osteoglycin
CLMP: CXADR like membrane protein
EGLN3: Egl-9 family hypoxia inducible factor 3
MFAP5: Microfibril associated protein 5
THBD: Thrombomodulin
DEGs: Differential expressed genes
ENO1: Enolase 1
EPGN: Epithelial mitogen
IL11: Interleukin 11
NRP2: Neuropilin 2
COL: Collagen
NGFR: Nerve growth factor receptor
ALDH1A3: Aldehyde dehydrogenase 1 family member A3
VEGFD: Vascular endothelial growth factor D
GDNF: Glial cell derived neurotrophic factor
HGF: Hepatocyte growth factor
BDNF: Brain derived neurotrophic factor
SEMA3G: Semaphorin 3G
IGFBP3: Insulin-like growth factor binding protein 3
HEY1: Hes related family bHLH transcription factor with YRPW motif 1
HBEGF: Heparin binding EGF like growth factor
OMD: Osteomodulin
EFEMP1: EGF containing fibulin extracellular matrix protein 1
CCL21: C–C chemokine ligand 21
LYVE1: Lymphatic vessel endothelial hyaluronan receptor 1
LIF: LIF interleukin 6 family cytokine
SELE: Selectin E
LTF: Lactotransferrin
CAMP: Cathelicidin antimicrobial peptide
GPI: Glucose-6-phosphate isomerase
AIMP1: Aminoacyl tRNA synthetase complex interacting multifunctional protein 1
EGR1: Early growth response 1
FOS: Fos proto-oncogene, AP-1 transcription factor subunit
LI1RN: Interleukin 1 receptor antagonist
TNFAIP6: TNF alpha induced protein 6
TNIP3: TNFAIP3 interacting protein 3
ACSL4: Acyl-CoA synthetase long chain family member 4
SMOX: Spermine oxidase
RPS18: Ribosomal protein S18
RPL18: Ribosomal protein L18
FRZB: Frizzled related protein
ATF3: Activating transcription factor 3
DCN: Decorin
MYL9: Myosin light chain 9
TNS1: Tensin 1
TMSB4X: Thymosin beta 4 X-linked
PFN1: Profilin 1
CST7: Cystatin F
TNFRSF1B: TNF receptor superfamily member 1B
IFITM3: Interferon induced transmembrane protein 3
THRB: Thyroid hormone receptor beta
FBLIM1: Filamin binding LIM protein 1
EIF2B3: Eukaryotic translation initiation factor 2B subunit gamma
TPM1: Tropomyosin 1
PKP1: Plakophilin 1
DCHS2: Dachsous cadherin-related 2
