# Supplementary material for: Single-cell transcriptomic analysis reveals differential cell subpopulations and distinct phenotype transition in normal and dissected ascending aorta
Source: Mol Med. 2022 Dec 19;28:158. doi: 10.1186/s10020-022-00584-4 (PMC9764678; doi:10.1186/s10020-022-00584-4)
Supplement: Supplementary file 1 — Additional file 1. Supplementary Figure I. Eight cell types in aortic tissues from control and ATAD group revealed by scRNA-seq. A, intraoperative identified ATAD and resected ascending aortic tissues. B, After washing by sterile PBS to remove residual blood and thrombus, ATAD samples were stored in preserving buffer for scRNA-seq. C, t-SNE plot exhibited all 14 clusters and 8 cell types identified in this study. D, the proportion of each cell type in control and ATAD group. The dashed line showed the boundary to discriminate the dominance of each cell type in control or ATAD group. E, the heatmap of marker genes for each cluster. F, t-SNE plots to show the expressions of representative marker genes for each cell type. Supplementary Figure II. The composition of VSMCs subpopulations in each sample and characteristics for each cluster of VSMCs. A, integrative t-SNE plot displayed the composition of VSMCs subpopulations for each sample. VSMCs was not identified from ATAD 5. B-C, t-SNE and violin plots showed the expressions of other representative marker genes. D, heatmap to identify functional modules of genes distinctly expressed in subpopulations of VSMCs. E-J, GO analysis for genes distinctly expressed in subpopulations of VSMCs that was not shown in Fig.2. Supplementary Figure III. The composition of FBs subpopulations in each sample and features for each cluster of FBs. A, integrative t-SNE plot showed the composition of each subpopulation of FBs in each sample. B-C, t-SNE and violin plots revealed the expression of other representative marker genes for subpopulations of FBs. D, heatmap to identify functional modules of genes distinctly expressed in subpopulations of FBs. E-F, GO analysis for genes distinctly expressed in subpopulations of FBs that was not shown in Fig.3. Supplementary Figure IV. The composition of ECs subpopulations in each sample and characteristics for each cluster of ECs. A, integrative t-SNE plot showed the composition of each subpopulation [file 10020_2022_584_MOESM1_ESM.zip › Supplementary materials-R.docx]

**Methods and materials**

**Single-cell Dissociation**

Single-cell RNA-seq experiment was performed by experimental personnel in the laboratory of NovelBio Bio-Pharm Technology Co.Ltd. The tissues were surgically removed and kept in MACS Tissue Storage Solution (Miltenyi Biotec) until processing. The tissue samples were processed as described below. Briefly, samples were first washed with phosphate-buffered saline (PBS), minced into small pieces (approximately 1mm^3^) on ice and enzymatically digested with 1mg/mL collagenase I (Worthington) for 60 min at 37°C, with agitation. After digestion, samples were sieved through a 70µm cell strainer, and centrifuged at 300g for 5 min. After the supernatant was removed, the pelleted cells were suspended in red blood cell lysis buffer (Miltenyi Biotec) to lyse red blood cells. After washing with PBS containing 0.04% BSA, the cell pellets were re-suspended in PBS containing 0.04% BSA and re-filtered through a 35μm cell strainer. Dissociated single cells were then stained with AO/PI for viability assessment using Countstar Fluorescence Cell Analyzer. The single-cell suspension was further enriched with a MACS dead cell removal kit (Miltenyi Biotec).

**Single-cell RNA Sequencing**

BD Rhapsody system was used to capture the transcriptomic information of the (9 sample-derived) single cells. Single-cell capture was achieved by random distribution of a single-cell suspension across >200,000 microwells through a limited dilution approach. Beads with oligonucleotide barcodes were added to saturation so that a bead was paired with a cell in a microwell. The cells were lysed in the microwell to hybridize mRNA molecules to barcoded capture oligos on the beads. Beads were collected into a single tube for reverse transcription and ExoI digestion. Upon cDNA synthesis, each cDNA molecule was tagged on the 5′ end (that is, the 3′ end of a mRNA transcript) with a unique molecular identifier (UMI) and cell barcode indicating its cell of origin. Whole transcriptome libraries were prepared using the BD Rhapsody single-cell whole-transcriptome amplification (WTA) workflow including random priming and extension (RPE), RPE amplification PCR and WTA index PCR. The libraries were quantified using a High Sensitivity DNA chip (Agilent) on a Bioanalyzer 2200 and the Qubit High Sensitivity DNA assay (Thermo Fisher Scientific). Sequencing was performed by illumina sequencer (Illumina, San Diego, CA) on a 150 bp paired-end run.

**Single-cell RNA Statistical Analysis**

scRNA-seq data analysis was performed by NovelBio Bio-Pharm Technology Co.,Ltd. with NovelBrain Cloud Analysis Platform. We applied fastp^1^ with default parameter filtering the adaptor sequence and removed the low quality reads to achieve the clean data. UMI-tools^2^ were applied for Single Cell Transcriptome Analysis to identify the cell barcode whitelist. The UMI-based clean data was mapped to human genome (Ensemble version 91) utilizing STAR mapping with customized parameter from UMI-tools standard pipeline to obtain the UMIs counts of each sample. Cells contained over 200 expressed genes and mitochondria UMI rate below 40% passed the cell quality filtering and mitochondria genes were removed in the expression table. Seurat package (version: 2.3.4, https://satijalab.org/seurat/) was used for cell normalization and regression based on the expression table according to the UMI counts of each sample and percent of mitochondria rate to obtain the scaled data. We used mutual nearest-neighbour (MNN) to remove potential batch effect. Subsequently, PCA was constructed based on the scaled data with top 2000 high variable genes and top 10 principals were used for tSNE construction and UMAP construction.

Utilizing graph-based cluster method (resolution = 0.8, recommended parameter), Since sample were processed and sequenced in batches, we acquired the unsupervised cell cluster result based the PCA top 10 principal and we calculated the marker genes by FindAllMarkers function with Wilcox rank sum test algorithm under following criteria:1. lnFC > 0.25, 2. P value < 0.05, 3. min.pct >0.1. In order to identify the cell type detailed, the clusters of same cell type were selected for re-tSNE analysis, graph-based clustering and marker analysis.

All processed single-cell RNA sequencing data in this study was deposited in GEO under the accession No. GSE189795.

**Differential Gene Expression Analysis**

To identify differentially expressed genes among samples, the function FindMarkers with Wilcox rank sum test algorithm was used under following criteria: 1. lnFC > 0.25, 2. P value < 0.05, 3. min.pct > 0.1.

**Co-regulated Gene Analysis**

To discover the gene co-regulation network, find_gene_modules function of monocle3^3^ was used with the default parameters.

**QuSAGE Analysis (Gene Enrichment Analysis)**

To characterize the relative activation of a given gene set such as pathway activation, contraction of vascular smooth muscle, collagen synthesis and cell cycle as described before, we performed QuSAGE^4^ (2.16.1) analysis.

**Immunohistochemistry and immunofluorescence**

All specimens were fixed with 4% paraformaldehyde (PFA) at 4℃ overnight, embedded with paraffin and 4μm sections were cut. All sections were subjected to antigen retrieval, endogenous peroxidase was blocked with 3% hydrogen peroxide, and non-specific binding sites were blocked with10% goat serum at room temperature for 1h. All slides were incubated with primary antibodies overnight at 4℃. Then, the slides were incubated with secondary antibodies at room temperature for 1h. 3, 3-diaminobenzidine (DAB) (K5007, Dako, Denmark) was added for 10min to visualize the proteins *in situ.* All pictures were captured by an optical microscope (Leica, Germany).

The fixed samples with PFA were dehydrated in 50% sucrose solution at 4℃. Then the samples were embedded in OCT and 7μm sections were cut in freezing microtome (Leica, Germany). 10% goat serum was used to block non-specific binding sites. Then primary antibodies were incubated with the samples overnight at 4℃. The primary antibodies were removed by washing, and the slides were incubated with secondary antibodies at room temperature for 1h and subsequently stained with 4',6-diamidino-2-phenylindole (DAPI) (KGA-215, Keygen, China) for 2min or DAB for 10min. The target proteins were visualized with laser confocal microscope (Leica, Germany).

In this study, MYH10 served as conserved synthetic phenotypic marker of aortic VSMCs. MYLK was chosen as the conserved contractile marker of VSMCs. DCN was selected as the marker of FBs. CD11b served as the marker of neutrophils. All antibodies used in this study was shown in Supplementary File V.

**CytoTRACE Analysis**

We applied CytoTRACE analysis for predicting differentiation state with default parameter^5^.

**Pseudo-Time Analysis**

We applied the Single-Cell Trajectories analysis utilizing Monocle2 (http://cole-trapnell-lab.github.io/monocle-release) using DDR-Tree and default parameter. Before Monocle analysis, we select marker genes of the Seurat clustering result and raw expression counts of the cell passed filtering. Based on the pseudo-time analysis, branch expression analysis modeling (BEAM Analysis) was applied for branch fate determined gene analysis.

**Cell Communication Analysis**

To enable a systematic analysis of cell–cell communication molecules, we applied cell communication analysis based on the CellPhoneDB^6^, a public repository of ligands, receptors and their interactions. Membrane, secreted and peripheral proteins of the cluster of different time point was annotated. Significant mean and Cell Communication significance (p-value<0.05) was calculated based on the interaction and the normalized cell matrix achieved by Seurat Normalization.

**Weighted Gene Co-expression Network Analysis**

The Weighted Gene Co-expression Network Analysis (WGCNA) R package^7^ was used for building signed co-expression networks for the subtypes of FBs, neutrophils and VSMCs. Biweight midcorrelation was first used to calculate pairwise correlations between genes. Next, pairwise topological overlap was calculated with a power of 23 based on a fit to scale-free topology. Co-expression modules comprised of positively correlated genes were then identified. The expression of each module was summarized by the module eigengene (ME, defined as the first principle component of all genes in a module), and Pearson correlations between MEs and different cell types were calculated.

**Reference**

1. Chen S, Zhou Y, Chen Y and Gu J. fastp: an ultra-fast all-in-one FASTQ preprocessor. *Bioinformatics*. 2018;34:i884-i890.

2. Smith T, Heger A and Sudbery I. UMI-tools: modeling sequencing errors in Unique Molecular Identifiers to improve quantification accuracy. *Genome Res*. 2017;27:491-499.

3. Cao J, Spielmann M, Qiu X, Huang X, Ibrahim DM, Hill AJ, Zhang F, Mundlos S, Christiansen L, Steemers FJ, Trapnell C and Shendure J. The single-cell transcriptional landscape of mammalian organogenesis. *Nature*. 2019;566:496-502.

4. Yaari G, Bolen CR, Thakar J and Kleinstein SH. Quantitative set analysis for gene expression: a method to quantify gene set differential expression including gene-gene correlations. *Nucleic Acids Res*. 2013;41:e170.

5. Gulati GS, Sikandar SS, Wesche DJ, Manjunath A, Bharadwaj A, Berger MJ, Ilagan F, Kuo AH, Hsieh RW, Cai S, Zabala M, Scheeren FA, Lobo NA, Qian D, Yu FB, Dirbas FM, Clarke MF and Newman AM. Single-cell transcriptional diversity is a hallmark of developmental potential. *Science (New York, NY)*. 2020;367:405-411.

6. Vento-Tormo R, Efremova M, Botting RA, Turco MY, Vento-Tormo M, Meyer KB, Park JE, Stephenson E, Polanski K, Goncalves A, Gardner L, Holmqvist S, Henriksson J, Zou A, Sharkey AM, Millar B, Innes B, Wood L, Wilbrey-Clark A, Payne RP, Ivarsson MA, Lisgo S, Filby A, Rowitch DH, Bulmer JN, Wright GJ, Stubbington MJT, Haniffa M, Moffett A and Teichmann SA. Single-cell reconstruction of the early maternal-fetal interface in humans. *Nature*. 2018;563:347-353.

7. Langfelder P and Horvath S. WGCNA: an R package for weighted correlation network analysis. *BMC Bioinformatics*. 2008;9:559.

**Supplementary figure legends**

**Supplementary Figure I**

Eight cell types in aortic tissues from control and ATAD group revealed by scRNA-seq. A, intraoperative identified ATAD and resected ascending aortic tissues. B, After washing by sterile PBS to remove residual blood and thrombus, ATAD samples were stored in preserving buffer for scRNA-seq. C, t-SNE plot exhibited all 14 clusters and 8 cell types identified in this study. D, the proportion of each cell type in control and ATAD group. The dashed line showed the boundary to discriminate the dominance of each cell type in control or ATAD group. E, the heatmap of marker genes for each cluster. F, t-SNE plots to show the expressions of representative marker genes for each cell type.

**Supplementary Figure II**

The composition of VSMCs subpopulations in each sample and characteristics for each cluster of VSMCs. A, integrative t-SNE plot displayed the composition of VSMCs subpopulations for each sample. VSMCs was not identified from ATAD 5. B-C, t-SNE and violin plots showed the expressions of other representative marker genes. D, heatmap to identify functional modules of genes distinctly expressed in subpopulations of VSMCs. E-J, GO analysis for genes distinctly expressed in subpopulations of VSMCs that was not shown in Fig.2.

**Supplementary Figure III**

The composition of FBs subpopulations in each sample and features for each cluster of FBs. A, integrative t-SNE plot showed the composition of each subpopulation of FBs in each sample. B-C, t-SNE and violin plots revealed the expression of other representative marker genes for subpopulations of FBs. D, heatmap to identify functional modules of genes distinctly expressed in subpopulations of FBs. E-F, GO analysis for genes distinctly expressed in subpopulations of FBs that was not shown in Fig.3.

**Supplementary Figure IV**

The composition of ECs subpopulations in each sample and characteristics for each cluster of ECs. A, integrative t-SNE plot showed the composition of each subpopulation of ECs in each sample. B, t-SNE and violin plots revealed the expression of other representative marker genes for subpopulations of ECs. C, heatmap to identify functional modules of genes distinctly expressed in subpopulations of ECs. D-I, GO analysis for genes distinctly expressed in subpopulations of ECs that was not shown in Fig.4.

**Supplementary Figure V**

The composition of neutrophils subpopulations in each sample and characteristics for each cluster of neutrophils. A, integrative t-SNE plot showed the composition of each subpopulation of neutrophils in all samples. B, t-SNE and violin plots revealed the expressions of other representative marker genes for subpopulations of neutrophils. C, heatmap to identify functional modules of genes distinctly expressed in subpopulations of neutrophils. D-I, GO analysis for genes distinctly expressed in subpopulations of neutrophils that was not shown in Fig.5.

**Supplementary Figure VI**

The composition of monocytes subpopulations in each sample and characteristics for each cluster of monocytes. A, integrative t-SNE plot showed the composition of each subpopulation of monocytes in all samples. B, t-SNE and violin plots revealed the expressions of other representative marker genes for subpopulations of monocytes. C, heatmap to identify functional modules of genes distinctly expressed in subpopulations of monocytes. D-J, GO analysis for genes distinctly expressed in subpopulations of monocytes.

**Supplementary Figure VII**

The composition of macrophages subpopulations in each sample and characteristics for each cluster of macrophages. A, integrative t-SNE plot showed the composition of each subpopulation of macrophages in all samples. B, t-SNE and violin plots revealed the expressions of other representative marker genes for subpopulations of macrophages. C, heatmap to identify functional modules of genes distinctly expressed in subpopulations of macrophages. D-H, GO analysis for genes distinctly expressed in subpopulations of macrophages.

**Supplementary Figure VIII**

Identification of T cell and NK cell as well as their expressions of marker genes.

**Supplementary Figure IX**

The composition of T cells in control and ATAD group as well as cell differentiation trajectory and gene expression alteration of FBs among the differentiation trajectory. A, t-SNE plot showed 5 subpopulations of T cells upon re-clustering. B, the proportion of each subpopulation in T cells. The dashed line discriminated the dominance of each subpopulation in control and ATAD group. C, heatmap of marker genes for each subpopulation of T cells. D, t-SNE and violin plots showed the expressions of representative marker genes for each subpopulation of T cells. E, the separated differentiation trajectory of FBs. F, the distribution of each subpopulation for FBs among the differentiation trajectory. G, alteration curves of other genes that was not shown in Fig.7. The full line represented cell fate 1, the dashed line represented cell fate 2. H-I, heatmap and curves of gene expression alteration relating to mRNA and translational processes. The full line represented cell fate 1, the dashed line represented cell fate 2. J-K, heatmap and curves of gene expression alteration relating to cell death and protein modification. The full line represented cell fate 1, the dashed line represented cell fate 2.

**Supplementary Figure X**

These bubble plots showed the interactions between VSMCs and subpopulations of FBs when FBs were selected as the origins of receptors.

**Supplementary Figure XI**

These bubble plots showed the interactions between VSMCs and subpopulations of neutrophils when they were selected as the origins of receptors.

**Supplementary Figure XII**

The cell differentiation trajectory and gene expression alteration of VSMCs and neutrophils. A, the separated cell differentiation trajectory for each subpopulation of VSMCs. B, the distribution for each subpopulation of VSMCs among the differentiation trajectory. C, heatmap of gene expression alteration relating to glycolysis in subpopulations of VSMCs. D, alteration curves of other genes in subpopulations of VSMCs. The full line represented cell fate 1, the dashed line represented cell fate 2. E-G, GO analysis for 3 clusters of altered genes in subpopulations of FBs. H, the separated cell differentiation trajectory for each subpopulation of neutrophils. I, the distribution for each subpopulation of neutrophils among the differentiation trajectory. J, heatmap of gene expression alteration relating to immune response and Th1 cell activation in subpopulations of neutrophils. K, alteration curves of other genes in subpopulations of neutrophils. The full line represented cell fate 1, the dashed line represented cell fate 2. L-N, GO analysis for 3 clusters of altered genes in subpopulations of neutrophils.

**Supplementary Figure XIII**

The networks and functions for gene modules identified by WGCNA among neutrophils, VSMCs and FBs. A-H, the networks and functions for gene modules identified by WGCNA that were not shown in Fig.8.

**Supplementary Figure XIV**

Overall expression of dissociation-induced IEGs in each subpopulation. A, correlation analysis among all subpopulations on the basis of top 2000 variable genes. B, overall expression of dissociation-induced IEGs in each subpopulation. The red label represented positive expression of dissociation-induced IEGs. The green label represented positive expression of dissociation-induced IEGs.

**Supplementary Figure XV**

Expressions of C1QA and C1QB in non-immune cells including VSMCs, FBs and ECs.

**Supplementary files**

**Supplementary file I**

Separated cell cluster and marker gene of each cell type for all samples in this study.

**Supplementary file II**

Patient demographics that were performed scRNA-seq.

**Supplementary file III**

Genes that were used to identify functions of VSMCs and FBs markers of neutrophils.

**Supplementary file IV**

The regulatory intensity results of CXCL12-ACKR3 between VSMCs 1 and FBs based on CellPhone database.

**Supplementary file V**

The gene list of dissociation-induced IEGs.

**Supplementary file VI**

The list of antibodies used in IHC and IF.
